# Akt inhibitor SC66 promotes cell sensitivity to cisplatin in chemoresistant ovarian cancer cells through inhibition of COL11A1 expression

**DOI:** 10.1038/s41419-019-1555-8

**Published:** 2019-04-11

**Authors:** Yi-Hui Wu, Yu-Fang Huang, Chien-Chin Chen, Cheng-Yang Chou

**Affiliations:** 1 Department of Obstetrics and Gynecology, National Cheng Kung University Hospital, College of Medicine, National Cheng Kung University, Tainan, Taiwan; 2 Department of Pathology, Chia-Yi Christian Hospital, Chia-Yi, Taiwan; 3 Department of Cosmetic Science, Chia Nan University of Pharmacy and Science, Tainan, Taiwan

## Abstract

We studied Akt inhibition using SC66 in a NOD-SCID xenograft mouse model and a panel of eight ovarian cancer cell lines. Elevated phospho-Akt levels in cancerous tissue were associated with short progression-free survival and overall survival. Cell sensitivity to SC66 was inversely correlated with phospho-Akt and COL11A1 expression levels, as well as resistance to cisplatin or paclitaxel. SC66 inhibited phosphorylation of Akt and its downstream effectors 4EBP1 and p70S6 kinase. SC66 also attenuated expression of TWIST1 and Mcl-1, factors important in cell invasiveness and anti-apoptosis, respectively. SC66-sensitized chemoresistant cells to cisplatin and paclitaxel treatment, and promoted apoptosis. In addition, SC66 inhibited COL11A1 expression via decreased binding of CCAAT/enhancer-binding protein beta (c/EBPβ), reducing chemoresistance and decreasing binding of nuclear transcription factor Y (NF-YA) to COL11A1. A mouse xenograft experiment demonstrated that SC66 treatment caused a reduction in tumor formation and enhanced the therapeutic efficacy of cisplatin. This study demonstrates the role of Akt in ovarian tumor progression and chemoresistance, and supports the application of SC66 as a therapy for ovarian cancer.

## Introduction

Epithelial ovarian carcinoma (EOC) is the most lethal gynecological malignancy^1^. The majority of patients are diagnosed at an advanced stage. Most patients initially respond to cytoreductive surgery and platinum-based chemotherapies; however, many eventually develop chemoresistant tumors, relapse, and die from the disease^2,3^. In addition, the incorporation of additional cytotoxic agents against ovarian cancer does not improve prognosis^4^. Therefore, to improve upon the current therapeutic options, there is a need to develop new interventions.

Akt, a key protein in the Akt/PI3K signaling pathway, is a serine/threonine protein kinase that, once activated by phosphorylation, plays an important role in the process of malignant transformation^5^. Phosphorylated Akt (p-Akt) is implicated in inducing signals that affect cell apoptosis and promote cellular proliferation and invasiveness through mammalian target of rapamycin (mTOR) activation^5–7^. Akt activation is a hallmark of a variety of human cancers^8,9^. Multiple mechanisms may lead to Akt activation in human cancers, among which the most frequent genetic alternations include loss of the tumor suppressor phosphatase and tensin homolog^10,11^ and mutational activation of the p110α catalytic subunit of phosphoinositide 3-kinase (PI3K)^12,13^. In addition, amplification of the genes encoding either Akt or PI3K^14,15^ and the constitutive activation of Akt have been observed in various human cancers^16,17^. Hyperactivation of Akt also occurs via deregulated signaling of many cell surface receptors, intracellular linkers, and signaling molecules, including the amplification/mutation of epidermal growth factor receptor/ErbB growth factor receptor family members and oncogenic mutations in the RAS family^18^. Moreover, Akt activation is associated with resistance to both chemotherapeutic agents and target agents^19^. Therefore, Akt inhibition may have therapeutic efficacy, either as monotherapy or in rational combination with other antitumor agents^20^.

COL11A1 belongs to the collagen family, which is the major component of the interstitial extracellular matrix. We previously investigated the importance of COL11A1 in EOC. Our results indicated that COL11A1 may promote cell aggressiveness via the transforming growth factor (TGF)-β1/Ets-1/matrix metalloproteinase-3 (MMP3) axis and the involvement of NF-YA-binding site in the *COL11A1* promoter^21^. We also elucidated the mechanisms by which COL11A1 promotes cancer cell sensitivity to anticancer drugs and we observed that, in ovarian cancer cells, chemoresistance developed via activation of the Akt/c/EBPβ pathway in concert with attenuated PDK1 ubiquitination and degradation^22^. In addition, COL11A1 reduced anticancer drug-induced apoptosis by upregulating TWIST1-mediated Mcl-1 expression^23^. These findings highlight the importance of COL11A1 in EOC tumor progression and chemoresistance, and suggest that targeting COL11A1 or Akt might provide new therapeutic opportunities in chemoresistant EOC.

We used GEO database through Connectivity Map website (http://www.broadinstitute.org/cMAP/) to find that SC66, an Akt inhibitor, may suppress COL11A1 (data not shown). SC66 is an allosteric inhibitor that facilitates Akt ubiquitination and deactivation through directly disrupting phosphatidylinositol (3,4,5)-triphosphate binding to pleckstrin homology domain^24^. SC66 has been demonstrated to promote cervical cancer cell death through inhibiting mTOR signaling^25^. In addition, SC66 in combination with doxorubicin and everolimus increases cell death and reduces tumor growth of hepatocellular carcinoma cells in mouse xenografts^26^. However, the mechanism by which SC66 modulates chemoresistance remains unclear. In the current study, we elucidated a novel molecular mechanism underlying the therapeutic action of SC66 in ovarian cancer cells, especially COL11A1-mediated chemoresistance.

## Results

### Cellular p-Akt expression in EOC patients

**Fig. 1:**
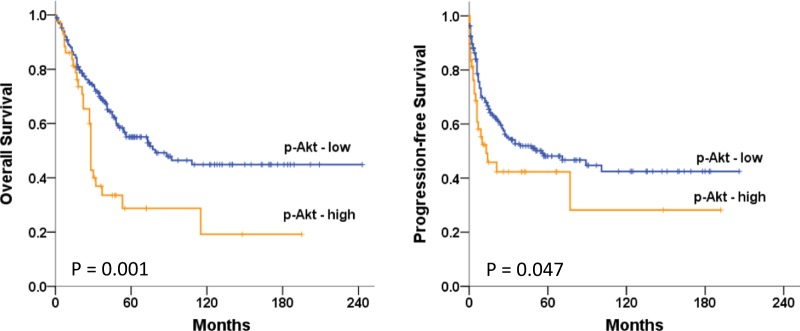
Five-year overall survival and progression-free survival. Kaplan–Meier curves of groups with p-Akt-low (*n* = 187) and p-Akt-high expression (*n* = 43) were demonstrated and were non-parametrically tested using the log-rank test

Tissue specimens and clinical data from 230 patients diagnosed with EOC were included in the study. During long-term follow-up, 110 patients (47.8%) developed progressive disease and 108 patients (47.0%) died. Associations between p-Akt expression in tumor tissue at the time of diagnosis and clinicopathological factors were examined. Cellular p-Akt overexpression was significantly associated with grade 3 tumors (*P* = 0.013) and cancer death (*P* = 0.021) (Supplementary Table 1). However, there was no correlation between cellular p-Akt expression and age, stage of disease, residual tumor size, or progression-free interval (PFI) ≤ 6 months. We did not observe statistically significant correlation between patient demographics and percentage of p-Akt-positive cells or immunostating score (intensity × percentage of p-Akt-positive cells, range 0–300). Long-term overall survival (OS) and progression-free survival (PFS) curves for the 230 patients are presented in Fig. 1. Patients with high p-Akt expression had significantly poorer OS and PFS than did patients with low p-Akt expression (*P* = 0.001 and *P* = 0.047, respectively).

### SC66 inhibits Akt signaling and expression of COL11A1, TWIST1, and Mcl-1 in ovarian cancer cells

**Fig. 2:**
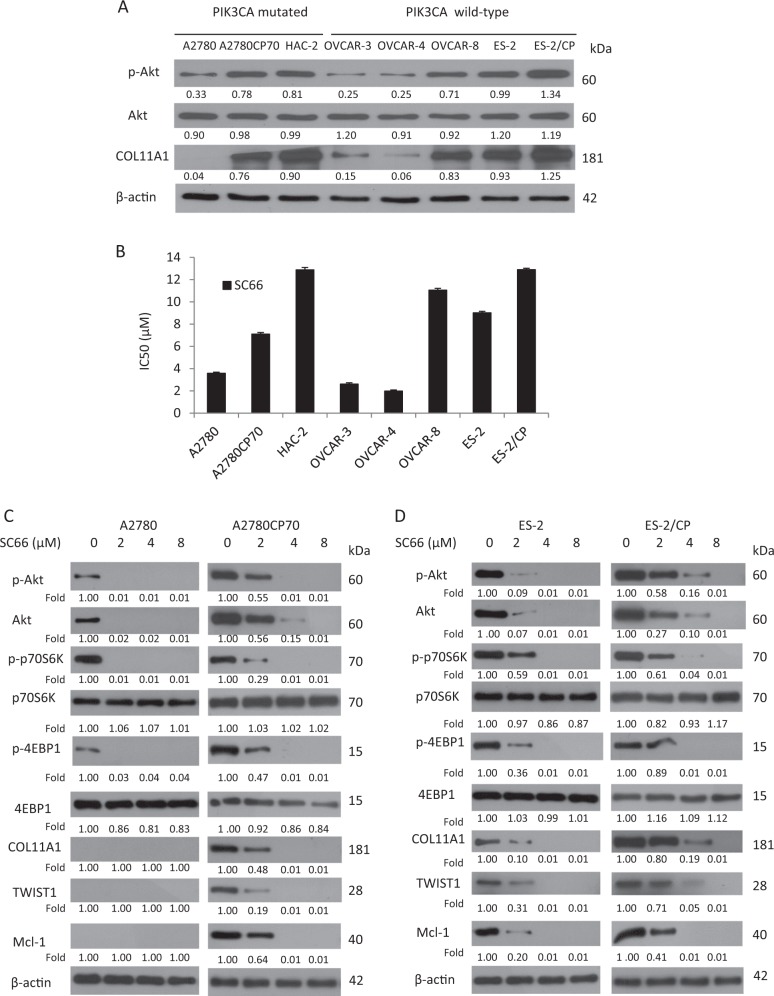
SC66 inhibits Akt signaling and expression of COL11A1, TWIST1, and Mcl-1 in ovarian cancer cells. **a** The protein expression levels of p-Akt, Akt, and COL11A1 in a panel of eight ovarian cancer cell lines were evaluated by western blotting. β-Actin protein was used as an internal loading control. All experiments were performed in triplicate. **b** The half maximal inhibitory concentration (IC_50_) value (mean ± SD) of SC66 in ovarian cancer cells was measured using the MTT assay. **c**, **d** The protein expression levels of p-Akt, Akt, p-p70S6K, p70S6K, p-4EBP1, 4EBP1, COL11A1, TWIST1, and Mcl-1 in cells treated with different concentrations of SC66 for 24 h were evaluated by western blotting. β-Actin was used as an internal loading control. All experiments were performed in triplicate

To examine the effect of SC66 on the proliferation of ovarian cancer cells in vitro, we performed MTT (3-(4,5-dimethylthiazol-2-yl)-2,5-diphenyltetrazolium bromide) assays, using a panel of eight human ovarian cancer cell lines, which included A2780, A2780CP70, OVCAR-3, OVCAR-4, and OVCAR-8, and clear cell type HAC-2, ES-2, and ES-2/CP. As shown in Fig. 2a, the expression levels of p-Akt and COL11A1 were low in A2780, OVCAR-3, and OVCAR-4 cells. In contrast, the expression levels of these factors were high in A2780CP70, HAC-2, OVCAR-8, ES-2, and ES-2/CP cells. The expression levels of p-Akt appeared to be independent of the status of the PIK3CA mutation (Fig. 2a). The expression levels of p-Akt and COL11A1 positively correlated with each other and were higher in chemoresistant A2780CP70 and ES-2/CP cells compared with those of their chemosensitive counterparts A2780 and ES-2 cells. Figure 2b shows a differential sensitivity of ovarian cells to SC66 treatment. A2780, OVCAR-3, and OVCAR-4 cells, which expressed low levels of p-Akt, were more sensitive to SC66 than were A2780CP70, HAC-2, OVCAR-8, ES-2, and ES-2/CP cells, which expressed high levels of p-Akt. These results suggested that cell sensitivity to SC66 inversely correlated with the expression level of p-Akt and COL11A1.

In addition to reduced p-Akt expression, SC66 treatment suppressed the expression of COL11A1, Akt, p-p70S6K, and p-4EBP1 at lower doses in chemosensitive A2780 (Fig. 2c, left panel) and ES-2 (Fig. 2d, left panel) cells. SC66 treatment was less effective at reducing the activation of the Akt pathway in chemoresistant A2780CP70 (Fig. 2c, right panel) and ES-2/CP (Fig. 2d, right panel) cells. We previously demonstrated that COL11A1-mediated nuclear factor-κB activation promoted the expression of TWIST1 and Mcl-1, factors associated with chemoresistance and anti-apoptosis in ovarian cancer cells^23^. Notably, expression of TWIST1 and Mcl-1 decreased in both chemosensitive and chemoresistant cells following SC66 treatment.

### SC66 sensitizes ovarian cancer cells to cisplatin and paclitaxel therapy

**Fig. 3:**
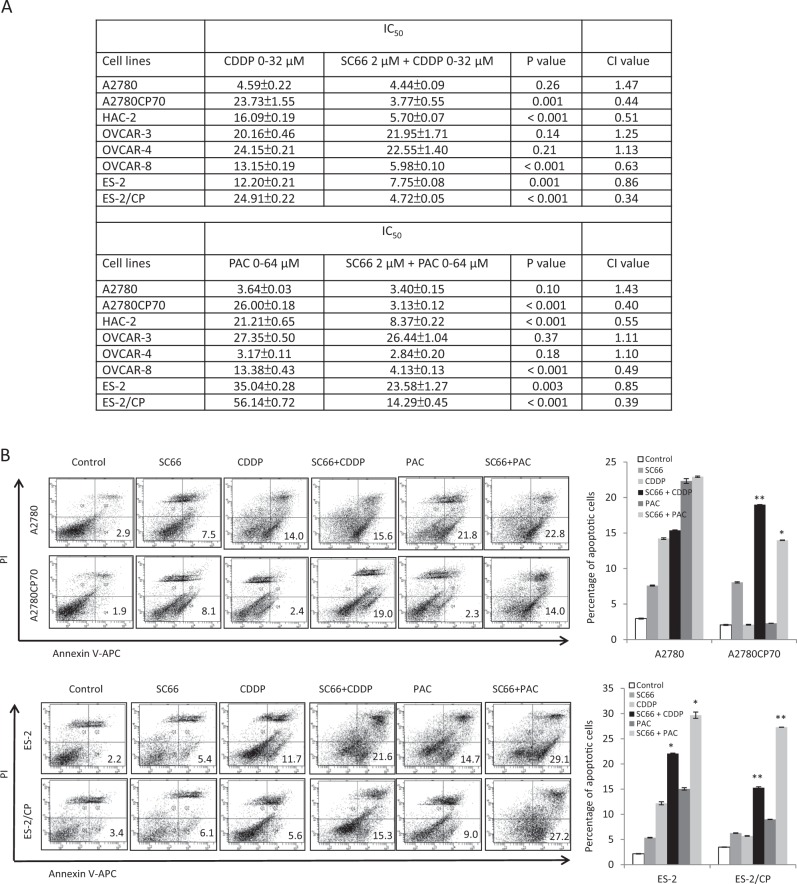

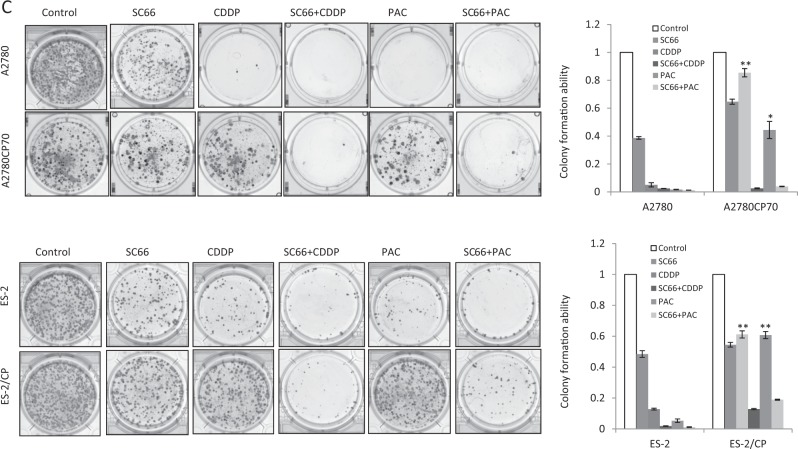
SC66 enhances the efficacy of anticancer drugs in ovarian cancer cells. **a** Ovarian cancer cells were treated with different concentrations of cisplatin (CDDP, 0–32 μM) or paclitaxel (PAC, 0–64 μM), or combined with 2 μM SC66 for 48 h. Each combination was tested with *n* = 5 replicates. After 48 h of treatment, cell viability was assessed by MTT assays. All experiments were performed in triplicate. The IC_50_ values of each agent in single or combination treatments and CI values of the SC66 + CDDP or SC66 + PAC combinations. *P*-value between the IC_50_ values of single vs. combination treatment. **b** Ovarian cancer cells were treated for 24 h with 2 μM SC66 alone or with the addition of anticancer drugs (10 μM) indicated. The percentage of apoptotic cells was determined by Annexin V and PI staining. Mean ± SD for three independent experiments are shown. *P < 0.05 and **P < 0.005, SC66 + CDDP vs. CDDP or SC66 + PAC vs. PAC. **c** Colony formation assay. Ovarian cancer cells were treated with 2 μM SC66 with or without the addition of the anticancer drugs (10 μM) indicated for 14 d (A2780 and A2780CP70) or 21 d (ES-2 and ES-2CP). After treatment, cells were stained with crystal violet. Mean ± SD for three independent experiments are shown. *P < 0.05 and **P < 0.005, SC66 + CDDP vs. CDDP or SC66 + PAC vs. PAC

We next determined whether SC66 enhanced the efficacy of anticancer drugs. The half maximal inhibitory concentration (IC_50_) values of each agent in single or combination treatments, as well as the combination index (CI) values of the SC66 + CDDP or SC66 + PAC combination, are listed in Fig. 3a. The CI indicated a synergistic cytotoxicity by combining SC66 with cisplatin or paclitaxel in HAC-2, OVCAR-8, and ES-2 cells, which demonstrated high expression levels of p-Akt and COL11A1. The synergistic cytotoxicity was also observed in chemoresistant A2780CP70 and ES-2/CP cells (Fig. 3a). Representative apoptotic profiles showed that increased apoptotic cell populations induced by combining SC66 and cisplatin or SC66 and paclitaxel were more apparent in chemoresistant cells (Fig. 3b). Consistent with these findings was the observation that treatment of chemoresistant cells with cisplatin or paclitaxel combined with SC66 resulted in a much stronger inhibitory effect on colony formation compared with that of SC66, cisplatin, or paclitaxel treatments alone (Fig. 3c). Together, these results demonstrated that SC66 sensitized chemoresistant cells to cisplatin and paclitaxel treatment and promoted apoptosis in these cells.

### SC66 regulates cell sensitivity to anticancer drugs and cell invasiveness via COL11A1 inhibition

**Fig. 4:**
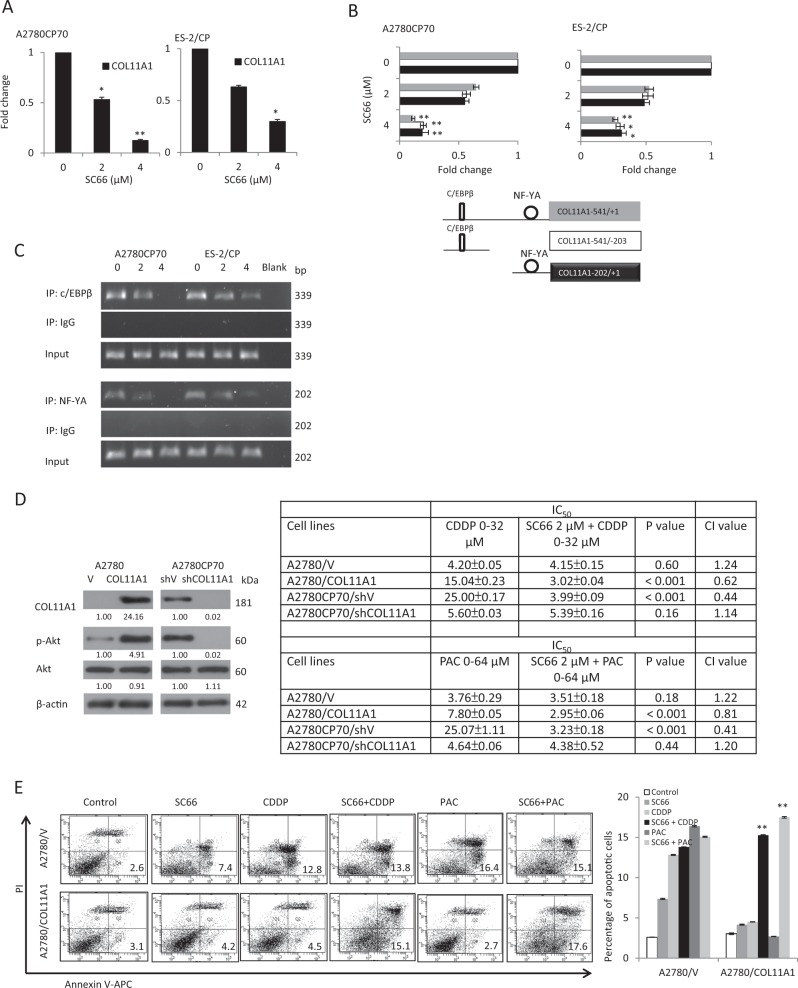

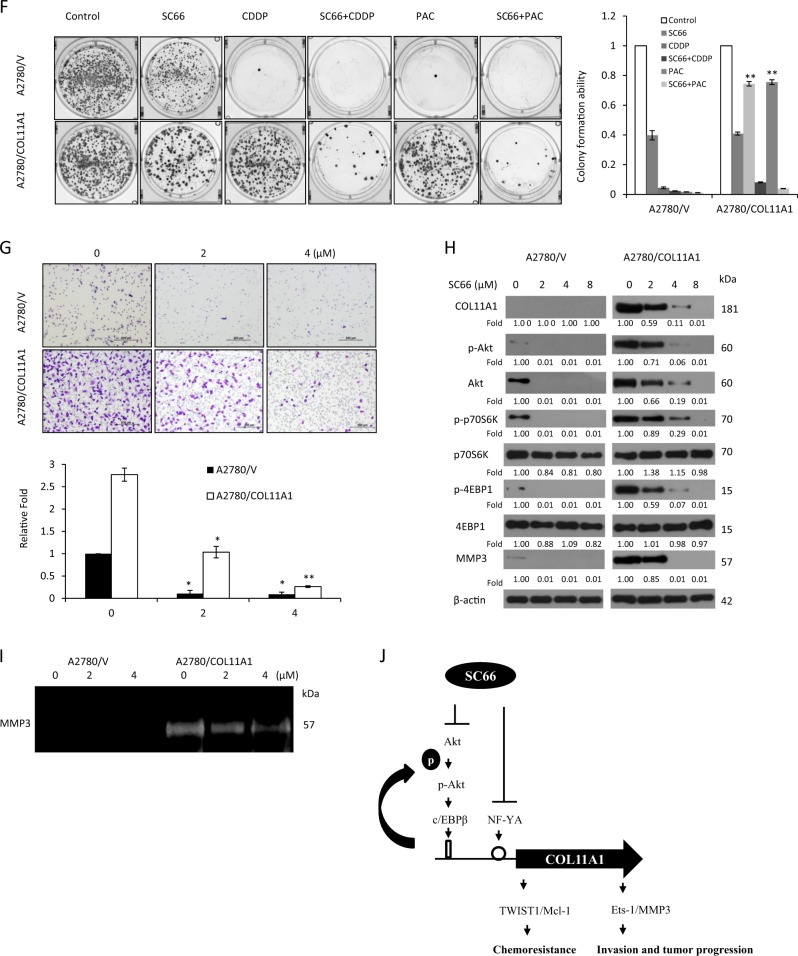
SC66 regulates cell sensitivity to anticancer drugs and cell invasiveness through inhibition of COL11A1. **a** A2780CP70 and ES-2/CP cells were treated with different concentrations of SC66 for 24 h and then COL11A1 expression was evaluated by real-time RT-PCR. All experiments were performed in triplicate. *P < 0.05 and **P < 0.005, SC66 vs. control. **b** A2780CP70 and ES-2/CP cells transfected with the *COL11A1* promoters were treated with different concentrations of SC66 for 24 h. Luciferase activity was measured and normalized to *Renilla* luciferase activity. All experiments were performed in triplicate. *P < 0.05 and **P < 0.005, SC66 vs. control. **c** ChIP assays were performed to evaluate c/EBPβ and NF-YA binding to the *COL11A1* promoter in A2780CP70 and ES-2/CP cells after treatment with different concentrations of SC66 for 24 h. **d** Left panel: Protein expression levels of COL11A1, p-Akt, and Akt in A2780 cells transfected with the COL11A1 plasmid and in A2780CP70 cells transfected with the shCOL11A1 plasmid were evaluated by western blotting. β-Actin protein was used as an internal loading control. Right panel: Ovarian cancer cells were treated with different concentrations of cisplatin (CDDP, 0–32 μM) or paclitaxel (PAC, 0–64 μM), or combined with 2 μM SC66 for 48 h. Each combination was tested with *n* = 5 replicates. After 48 h, cell viability was assessed by MTT assays. All experiments were performed in triplicate. The IC_50_ values of each agent in single or combination treatments and CI values of the SC66 + CDDP or SC66 + PAC combinations. *P*-value between the IC_50_ values of single vs. combination treatment. **e** A2780/V and A2780/COL11A1 cells were treated for 24 h with 2 μM SC66 alone or with the addition of anticancer drugs (10 μM) indicated. The percentage of apoptotic cells was determined by Annexin V and PI staining. Mean ± SD for three independent experiments are shown. **P < 0.005, SC66 + CDDP vs. CDDP or SC66 + PAC vs. PAC. **f** Colony formation assay. A2780/V and A2780/COL11A1 cells were treated with 2 μM SC66 alone or with the addition anticancer drugs (10 μM) as indicated for 14 d. After treatment, cells were stained with crystal violet. Mean ± SD for three independent experiments are shown. **P < 0.005, SC66 + CDDP vs. CDDP or SC66 + PAC vs. PAC. **g** Invasion activity in vitro of A2780/V and A2780/COL11A1 cells after treatment with different concentrations of SC66 for 24 h. All data represent the mean ± SD of three separate experiments. *P < 0.05 and **P < 0.005, SC66 vs. control. **h** Protein expression levels of COL11A1, p-Akt, Akt, p-p70S6K, p70S6K, p-4EBP1, 4EBP1, and MMP3 in A2780/V and A2780/COL11A1 cells treated with different concentrations of SC66 for 24 h were evaluated by western blotting. β-Actin was used as an internal loading control. All experiments were performed in triplicate. **i** MMP3 activity was evaluated by casein zymography in A2780/V and A2780/COL11A1 cells treated with different concentrations of SC66 for 24 h. All experiments were performed in triplicate. **j** A model illustrating the hypothetical role of SC66 in controlling COL11A1-mediated chemoresistance and invasiveness in ovarian cancer cells

As previously mentioned, COL11A1, TWIST1, and Mcl-1 expression was decreased by SC66 treatment (Fig. 2c, d). Our previous findings showed that COL11A1 confers resistance to cisplatin and paclitaxel in ovarian cancer cells via increased Akt phosphorylation^22^, and that COL11A1 regulates TWIST1 and Mcl-1 to induce chemoresistance and inhibit apoptosis^23^. Thus, we hypothesized that SC66 enhances cell sensitivity to anticancer drugs through COL11A1 regulation. To confirm that COL11A1 transcription was regulated by SC66, A2780CP70 and ES-2/CP cells were treated with SC66 for 24 h. Our data show that COL11A1 RNA expression was reduced by SC66 in both cells (Fig. 4a). To further explore the mechanism by which SC66 treatment regulated *COL11A1* transcription, a COL11A1 fragment (−541 to −1) was amplified by PCR, sequenced, and cloned into a luciferase reporter plasmid. Therefore, a series of *COL11A1* promoter constructs containing various deletions (Fig. 4b) was then constructed and constructs were individually transiently transfected into A2780CP70 and ES-2/CP cells. These cells were then treated with SC66 for 24 h. The luciferase activity of the transfectants containing the *COL11A1*−541/+1, *COL11A1*−541/−203, and *COL11A1*−202/+1 promoter fragments was significantly decreased by SC66 treatment in a dose-dependent manner. Our previous reports indicate that the c/EBPβ-binding site and NF-YA-binding site are located in the −541/−203 and −202/+1 regions, respectively, of the *COL11A1* promoter^21,22^. Chromatin immunoprecipitation (ChIP) analysis confirmed that SC66 treatment reduced NF-YA binding and c/EBPβ binding to the *COL11A1* promoter region in both cells (Fig. 4c). These results indicated that COL11A1 was regulated by SC66 in ovarian cancer cells.

We further verified whether SC66 would overcome COL11A1-mediated chemoresistance in ovarian cancer cells. A *COL11A1* cDNA plasmid was introduced into low COL11A1-expressing A2780 cells to induce the overexpression of COL11A1, whereas a small interfering RNA (siRNA) specific for the *COL11A1* gene (shCOL11A1) was introduced into A2780CP70 cells to knock down COL11A1 expression. As expected, p-Akt and COL11A1 expression was increased in COL11A1-overexpressing A2780 cells and decreased in COL11A1-knockdown A2780CP70 cells (Fig. 4d, left panel). The CI showed a synergistic cytotoxicity of SC66 with anticancer drugs in A2780/COL11A1 and A2780CP70/shV cells. In contrast, the synergistic cytotoxicity was not observed in A2780/V or A2780CP70/shCOL11A1 cells (Fig. 4d, right panel). Further experiments displayed significant synergistic effects in A2780/COL11A1 cells (CI_50_ = 0.78, *P* < 0.001 for SC66 + CDDP vs. CDDP; CI_50_ = 0.77, *P* = 0.002 for SC66 + PAC vs. PAC, Supplementary Fig. 1), but not in A2780/V cells. Representative apoptotic profiles further indicated that the combination treatment of A2780/COL11A1 cells with SC66 and cisplatin or paclitaxel increased apoptotic cell populations (Fig. 4e). In agreement with these results, the combined treatment of chemoresistant cells with SC66 and cisplatin or paclitaxel resulted in a much stronger inhibitory effect on colony formation compared with SC66, cisplatin, or paclitaxel treatments alone (Fig. 4f). Together, these results demonstrated that SC66 treatment sensitized cells to cisplatin and paclitaxel treatment, and promoted apoptosis by inhibiting COL11A1 activation.

Our previous report also indicated that COL11A1 promotes tumor aggressiveness via the TGF-β1–MMP3 axis, and that a NF-YA-binding site on the *COL11A1* promoter is the major determinant of TGF-β1-dependent COL11A1 activation^21^. We next examined whether SC66 treatment would decrease COL11A1-mediated cell invasiveness via the inhibition of MMP3. As shown in Fig. 4g, cell invasion ability was increased in A2780/COL11A1 cells compared with that of A2780/V cells, and the increased invasiveness was inhibited by the addition of SC66. Western blotting analysis showed that the elevated expression levels of COL11A1, Akt, p-Akt, and MMP3 in A2780/COL11A1 cells were inhibited by SC66 treatment (Fig. 4h). MMP3 activity, as measured by casein zymography, was decreased by 4 μM SC66 treatment (Fig. 4i). Taken together, SC66 treatment regulated the sensitivity of cells to anticancer drugs and cell invasiveness may be mediated through the inhibition of COL11A1 (Fig. 4j).

### SC66 enhances anticancer drug therapy in mouse xenografts

**Fig. 5:**
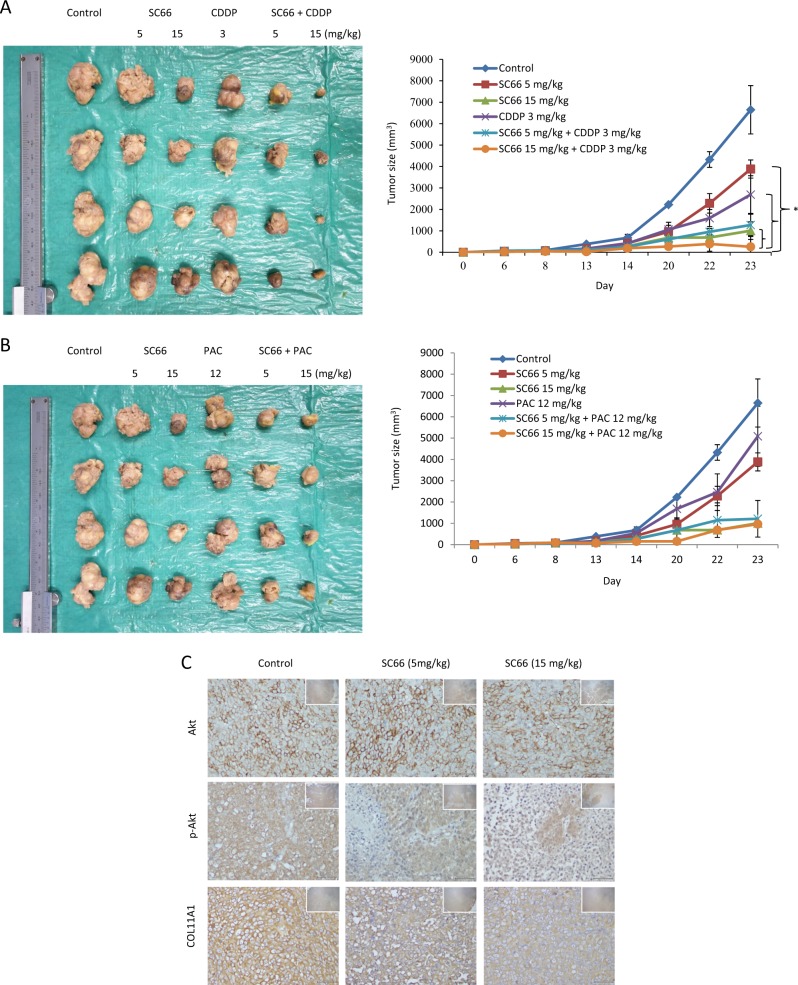
SC66 increases the sensitivity of mouse xenografts to anticancer drugs. **a** A2780/COL11A1 ovarian cancer xenografts treated with only SC66 at doses of 5 mg/kg and 15 mg/kg or combined with 3 mg/kg cisplatin. * *P* < 0.05, on Day 23, SC66 15 mg/kg + CDDP 3 mg/kg vs. CDDP alone (*P* = 0.021) or varying doses of SC66 (5 mg/kg, *P* = 0.021; 15 mg/kg, *P* = 0.043). **b** A2780/COL11A1 ovarian cancer xenografts treated with only SC66 at doses of 5 and 15 mg/kg or combined with 12 mg/kg paclitaxel. CDDP: cisplatin; PAC: paclitaxel. Tumor size (mean ± SD): control group 6648.38 ± 2258.12 mm^3^; 5 mg/kg SC66 group 3883.70 ± 843.91 mm^3^; 15 mg/kg SC66 group 1006.93 ± 819.18 mm^3^; CDDP group 2691.58 ± 1756.27 mm^3^; 5 mg/kg SC66 + 3 mg/kg CDDP group 995.83 ± 519.68 mm^3^; 15 mg/kg SC66 + 3 mg/kg CDDP group 256.30 ± 90.52 mm^3^; PAC group 5086.15 ± 2816.66 mm^3^; 5 mg/kg SC66 + 12 mg/kg PAC group 1143.63 ± 291.24 mm^3^; 15 mg/kg SC66 + 12 mg/kg PAC group 964.13 ± 853.27 mm^3^. **c** Representative IHC photos of p-Akt, Akt, and COL11A1 in ovarian tumor samples from mice treated with SC66 or vehicle controls

To determine whether SC66 could suppress tumor growth in vivo, mice were subcutaneously injected with 1 × 10^6^ A2780/COL11A1 cells and treated with an intraperitoneal injection of SC66, with or without cisplatin (Fig. 5a) and paclitaxel (Fig. 5b). Tumor formation was not observed in mice injected with A2780/V cells (Supplementary Fig. 2). When compared with the treatment vehicle controls, a single treatment with 3 mg/kg cisplatin (*P* = 0.021) or varying doses of SC66 (5 mg/kg, *P* = 0.043; 15 mg/kg, *P* = 0.021) significantly inhibited tumor growth in mouse xenografts on Day 23. In addition, tumor size was significantly reduced in mice treated with 15 mg/kg SC66 + 3 mg/kg cisplatin compared with that in mice treated with either 3 mg/kg cisplatin alone (*P* = 0.021) or SC66 alone (5 mg/kg, *P* = 0.021; 15 mg/kg, *P* = 0.043). In contrast, the absolute tumor size was reduced in mice treated with 12 mg/kg paclitaxel compared with that in mice treated with vehicle, but the difference did not reach statistical significance (*P* = 0.564). Similarly, the difference in tumor size failed to reach statistical significance when comparing the treatment of xenograft mice with 5 mg/kg SC66 + 12 mg/kg paclitaxel or 15 mg/kg SC66 + 12 mg/kg paclitaxel compared with that of mice treated with only 12 mg/kg paclitaxel (*P* = 0.083 and *P* = 0.083, respectively). The p-Akt and COL11A1 protein levels were decreased in cancerous tissues of mice treated with SC66 compared with that of vehicle controls in which Akt expression did not change (Fig. 5c). Ki-67 expression was significantly reduced in mice treated with 15 mg/kg SC66 + 3 mg/kg cisplatin and 15 mg/kg SC66 + 12 mg/kg paclitaxel compared with that in mice treated with 3 mg/kg cisplatin alone and 12 mg/kg paclitaxel alone, respectively (*P* = 0.021 for cisplatin; *P* = 0.020 for paclitaxel, Supplementary Fig. 3A). The expression of cleaved caspase 3 was significantly increased in mice treated with 15 mg/kg SC66 + 3 mg/kg cisplatin and 15 mg/kg + 12 mg/kg paclitaxel compared with that in mice treated with 3 mg/kg cisplatin alone and 12 mg/kg paclitaxel alone, respectively (*P* = 0.019 for cisplatin; *P* = 0.021 for paclitaxel, Supplementary Fig. 3B). The body weight of animals receiving cisplatin, paclitaxel, or SC66, alone or in combination, remained relatively unchanged, suggesting a negligible level of toxicity, if any, caused by the treatments (data not shown).

## Discussion

In the current study, EOC patients with tumors overexpressing p-Akt had shorter PFS and OS, and higher rates of cancer death, which indicated that elevated p-Akt expression was an unfavorable tumor biomarker of long-term survival. Furthermore, our findings from the mouse xenograft model reflected an inhibitory effect of the Akt inhibitor SC66 on tumor formation and cell survival, and suggested that SC66 treatment sensitized cancer cells to cisplatin chemotherapy. These in vivo results were reinforced by the in vitro findings from a panel of eight ovarian cancer cell lines in which SC66 treatment suppressed cell proliferation and invasion, and regulated COL11A1 to overcome chemoresistance and promote cell apoptosis. The regulation of COL11A1 was through the dual suppression of c/EBPβ and NF-YA binding to the *COL11A1* promoter.

The p-Akt has been implicated in inducing signals that affect cell apoptosis and promote cell proliferation and invasiveness through the crucial mechanism of mTOR activation^27^. Overexpressed p-Akt is associated with a poor prognosis of some human cancers^28–31^. For instance, studies of p-Akt expression in ovarian cancer have shown that p-Akt is a marker for a poor prognosis^32–34^. In contrast, one study demonstrated no significant association between p-Akt and OS^35^. In the current study, more patients with high p-Akt levels were allocated in the group of clinically defined chemoresistance, although the difference did not achieve statistical significance. Our results showed that patients with tumors overexpressing p-Akt had a poorer survival rate and the overexpression was associated with high-grade tumors and death. Overall, p-Akt overexpression may be a common prognostic factor shared by multiple types of human cancers and thus has the potential for being a therapeutic target of clinical significance.

The PI3K/Akt signaling pathway has become the focus of interest as a critical regulator of tumor cell survival and a number of Akt pathway inhibitors have been identified with a wide variety of potencies and specificities^36,37^. SC66, an inhibitor of Akt and mTOR, effectively induces apoptosis in cervical^24^ and hepatoma carcinoma cells^25^. SC66 promotes cell death in cervical cancer cells through disruption of Akt signaling and glucose uptake^24^, and SC66 exerts its antitumor effects on hepatoma cells by the production of reactive oxygen species (ROS), induction of anoikis-mediated cell death, and inhibition of the Akt cell survival pathway^25^. Akt is phosphorylated via crosstalk with Ras and regulates cell proliferation and chemoresistance^38^. In the current study, we described the in vitro and in vivo effects of SC66 on EOC. In addition to suppression of Akt/mTOR signaling, our data revealed a novel molecular mechanism underlying SC66-induced cytotoxicity. In brief, SC66 regulated COL11A1, thereby enhancing the sensitivity of cells to anticancer drugs, suppressing cell proliferation and invasion, and promoting apoptosis through the dual suppression of c/EBPβ and NF-YA binding to the *COL11A1* promoter. Notably, similar concentrations of SC66 caused less cytotoxicity in normal ovarian cells compared with that in cancerous cells, suggesting that SC66 preferentially killed malignant cells (Supplementary Fig. 4).

Chemoresistance often results in patient death, due to a lack of effective treatment. Cusimano et al.^25^ reported that SC66 in combination with doxorubicin and everolimus in HCC cells effectively reduces cell viability. Lin et al.^39^ described that the Akt inhibitor MK-2206 enhances the efficacy of cisplatin and paclitaxel in vitro, in both Akt-active and Akt-inactive ovarian cancer cells, but through different mechanisms that include the inhibition of Akt signaling, induction of ROS, and restoration of p53 levels. Based on our in vitro findings, SC66 enhanced the efficacy of cisplatin and paclitaxel, in agreement with the results from the MK-2206 studies^39^. However, our results showed that COL11A1 mRNA expression and promoter activity was regulated by SC66 (Fig. 4a, b), but not by MK-2206 (Supplementary Fig. 5A and 5B). We also found out that the expression of PDK1, well known as the kinase responsible for the phosphorylation and activation of Akt^22^, was inhibited by SC66, but not by MK-2206 (Supplementary Fig. 5C). Our results suggest that Akt inhibitors might exert their inhibition of Akt signaling through different mechanisms. It has been showed that SC66 promotes Akt ubiquitination^25^, whereas MK-2206 inhibits Akt phosphorylation^39^. Further investigation is required to explore the precise molecular mechanisms underlying Akt inhibitors-regulated Akt-related signaling. Our in vivo results from mouse xenografts indicated that SC66 sensitized cancer cells to cisplatin therapy more efficaciously than did the combined use of SC66 and paclitaxel. Further investigation is required to determine the best combinations of SC66 and cytotoxic agents or other anticancer agents. Our previous report indicated that chemoresistance in ovarian cancer cells develops through activation of the Akt/c/EBPβ pathway in concert with increased degradation of PDK1. The c/EBPβ-binding site on the *COL11A1* promoter (−541/−203) region has been identified as the major determinant of anti-cancer drug-induced COL11A1 expression^22^. In addition, COL11A1 interferes with anti-cancer drug-induced apoptosis in ovarian cancer cells by upregulating TWIST1-mediated Mcl-1 expression^23^. In the current study, we provide the first evidence that SC66 regulates cell sensitivity to cisplatin and paclitaxel, and cell apoptosis through inhibiting COL11A1 expression via decreased binding of c/EBPβ to the *COL11A1* promoter (Fig. 4d, e), and thus downregulated the expression of TWIST1 and Mcl-1.

In addition to promoting apoptosis, we also showed that SC66 inhibits invasiveness in ovarian cancer cells. Our previous report indicated that the NF-YA-binding site on the *COL11A1* promoter (−202/+1) is critical for COL11A1 activation^21^. In the present study, SC66 inhibited NF-YA binding to the *COL11A1* promoter (Fig. 4d, e) and decreased MMP3 protein expression and activity (Fig. 4g, h). Together, our results suggest the possibility that SC66 inhibits invasiveness of ovarian cancer cells through the TGF-β1/Ets-1/MMP3 axis. However, overexpression of TWIST1 is not only linked to resistance to apoptosis^23,40–43^ but also to increased cell migration, invasion, and metastasis^44–46^. Previous findings indicate that TWIST1 promotes invasion via the upregulation of MMP1 in human melanoma cells^47^. Thus, further investigation is required to explore whether MMPs, other than MMP3, may be implicated in SC66 therapy.

In conclusion, we examined p-Akt in cancerous tissues in regard to showing poor prognosis for patients with EOC. Furthermore, combination treatment, particularly using cisplatin and SC66, was more effective in inhibiting tumor growth in mouse xenografts than was treatment with a single agent. Finally, we evaluated the relationship of SC66 treatment with COL11A1 and revealed a novel mechanism for COL11A1 regulation by SC66 resulting in the sensitization of cancer cells to chemotherapy and the promotion of tumor cell apoptosis in EOC. We suggest that SC66 may have potential for use in patients with COL11A1-positive ovarian cancer.

## Materials and methods

### Study population

A total of 230 ovarian cancer patients with stage I–IV EOC, according to the International Federation of Gynecology and Obstetrics cancer staging system, who underwent comprehensive staging surgery or cytoreduction at the National Cheng Kung University Hospital between 2002 and 2010 were enrolled in the study. A review of the medical records and pathology slides for these patients was the source of information regarding the clinical characteristics, pathological diagnoses, and outcomes. Patients were followed after treatment, with the date of the latest record retrieved being 31 January 2015. Both OS and PFS were calculated based on the date of diagnosis, and the PFI was determined using the date of last contact. EOC patients with PFI ≤ 6 months were categorized as “resistant” to platinum-based chemotherapy and those with PFI > 6 months were categorized as “sensitive” to platinum-based chemotherapy. The investigation was approved by the National Cheng Kung University Hospital institutional review board (A-ER-105-017) and the experiments were undertaken with the understanding and written consent of each patient. The study methodologies in accordance with the standards set by the Declaration of Helsinki.

### Evaluation of p-Akt levels by immunohistochemistry

Ovarian cancer tissue sections were prepared as previously described^21^. Formalin-fixed paraffin-embedded tissue sections were deparaffinized and stained for p-Akt protein using a standard automated IHC slide staining system (BenchMark XT autostainer; Ventana Medical Systems, Tucson, AZ, USA) after microwave-enhanced epitope retrieval. The anti-p-Akt (Ser473) primary antibody (4060S) was purchased from Cell Signaling Technology (Danvers, MA, USA) and applied at a dilution of 1:100. Negative controls were treated with phosphate-buffered saline (PBS). High-expressing p-Akt-positive human lung carcinoma tissues were used as positive controls. The investigator evaluating the IHC experiments (C.-C.C., a gynecologic pathologist) was blinded to the patient clinical outcome data. Staining intensity was categorized as negative (grade 0), weak (grade 1), moderate (grade 2), or strong (grade 3). A grade 0–2 in staining intensity was designated as low expression, whereas a grade 3 was considered as high expression (Supplementary Fig. 6).

### Cells and media

The human ovarian cancer cell lines A2780 and A2780CP70 were obtained from the American Type Culture Collection (Manassas, VA, USA). The HAC-2 cell line was obtained from the Japanese Collection of Research Bioresources Cell Bank (Osaka, Japan). The OVCAR-3, OVCAR-4, and OVCAR-8 cell lines were purchased through the National Cancer Institute DTP tumor repository program (Frederick, MD, USA). The ES-2 cell line was purchased from the Bioresource Collection and Research Center of the Food Industry Research and Development Institute (Hsinchu, Taiwan). Cisplatin-resistant strains of ES-2 (ES-2/CP) were developed in our laboratory as previously described^48^. Immortalized ovarian surface epithelial cells (HOSE 6-3 and HOSE 11-12) were kindly provided by Dr George Tsao (Department of Anatomy, The University of Hong Kong). Cell lines A2780, A2780CP70, OVCAR-3, OVCAR-4, and OVCA-8 were grown in RPMI-1640 medium supplemented with 10% fetal bovine serum (FBS). HAC-2 cells were growth in Minimal Essential Medium supplemented with 15% FBS. ES-2 and ES-2/CP cells were grown in Mycos 5A medium supplemented with 10% FBS. HOSE 6-3 and HOSE 11-12 cells were grown in 1:1 MCDB105/M199 media supplemented with 15% FBS. All cells were grown at 37 °C in a 5% carbon dioxide atmosphere. Cells were cultured and stored according to the supplier’s instructions and used between passage 5 and 20. Once thawed, the cell lines were routinely authenticated approximately every 6 months, with the cells last being tested in March 2018, by cell morphology monitoring, growth curve analysis, species verification by iso-enzymology and karyotyping, identity verification using short tandem repeat-profiling analysis, and contamination checks.

### COL11A1 knockdown and transfection

The siRNAs directed against human COL11A1 (COL11A1 shRNA, sc-72956-SH, pools of three target-specific 19–25 nt siRNAs) and the non-targeting negative control shRNA plasmid (sc-108060-SH) were purchased from Santa Cruz Biotechnology, Inc. (Dallas, TX, USA). To establish stable clones, the COL11A1 knockdown plasmids were transfected into A2780CP70 cells using the Lipofectamine transfection reagent (Invitrogen). Twenty-four hours after transfection, stable transfectants were selected in G418 (Sigma) at a concentration of 800 μg/mL. Thereafter, the selection medium was replaced every 3 days. After 2 weeks of selection in G418, clones of resistant cells were isolated and allowed to proliferate in medium containing G418 at 800 μg/mL.

### COL11A1 overexpression and transfection

COL11A1 cDNA (BC117697 GE Healthcare) was cloned into the pCMV6-AC-GFP vector (PS100010 OriGene). Plasmids were transfected into ovarian cancer cells using the HyFect^TM^ DNA transfection reagent (Leadgene Biomedical, Taiwan), according to the manufacturer’s protocol. To establish stable clones, COL11A1 expression plasmids were transfected into A2780 cells and stable transfectants were selected 24 h later in G418 (Sigma) at a concentration of 400 μg/mL. Thereafter, the selection medium was replaced every 3 d. After 2 weeks of selection in G418, clones of resistant cells were isolated and allowed to proliferate in medium containing G418 at 400 μg/mL.

### Western blotting analysis

Proteins were extracted and equal amounts were separated by 8–15% sodium dodecyl sulfate (SDS)-polyacrylamide gel electrophoresis, as previously described^21^.

### Antibodies and reagents

Antibodies specific for Akt (9272 for western blotting), phospho-Akt (ser473, 9271), phospho-p70S6K (9205), P70S6K (9202), phospho-4EBP1 (9451), 4EBP1 (9452), caspase 3 (9664), mouse IgG (7076), and rabbit IgG (7074) were obtained from Cell Signaling Technology. An anti-Akt1 antibody for immunohistochemistry (IHC; A11027) was purchased from ABclonal (Woburn, MA, USA). An anti-Ki-67 (61-0078) antibody was purchased from Genemed (South San Francisco, CA, USA). An anti-COL11A1 antibody (sc-68853 for IHC), anti-TWIST1 (sc-15393), anti-Mcl-1 (sc-12756), anti-NF-YA (sc-10779), anti-c/EBPβ sc-150 goat anti-rabbit IgG-HRP (sc-2005), goat anti-rabbit IgG-HRP (sc-2054), and anti-β-actin (sc-47778) antibodies were purchased from Santa Cruz Biotechnology. An antibody against COL11A1 (GTX55142 for western blotting) was obtained from GeneTex (Irvine, CA, USA). An antibody against MMP3 (ab38912) was obtained from Abcam (Cambridge, UK). The Akt inhibitor SC66 was purchased from Cayman Chemicals (Ann Arbor, MI, USA). Cisplatin (Fresenius Kabi Oncology, Ltd) and paclitaxel (Corden Pharma Latina S.P.A.) were provided by the Cancer Center of National Cheng Kung University Hospital.

### Calculation of IC_50_ and CI analysis

Cisplatin (10 mM) and paclitaxel (10 mM) were dissolved in distilled water, whereas SC66 (10 mM) stock solutions were prepared in dimethylsulfoxide and were stored at −20 °C. When the experiment was carried out, the culture medium was used for drug dilution. Cells were exposed to varying concentrations of cisplatin (0–32 μM), paclitaxel (0–64 μM), or SC66 (0–22 μM) for 48 h. The in vitro cytotoxic effects of these treatments were determined using an MTT assay (at 570 nm) and cell viability was expressed as a percentage of the viability of control cells (% of control). IC_50_ values were determined from dose–response curve of percent growth inhibition against test concentrations. For combination treatment, cells co-treated with 2 μM SC66 and different concentration of cisplatin (0–32 μM) or paclitaxel (0–64 μM) for 48 h. CI analysis is the most common method used in evaluating the nature of drug interactions in combination chemotherapy and provides useful quantitative information^26^. CI is a numerical value calculated according to the following formula: CI = C_A,X_/IC_X,A_ + C_B,X_/IC_X,B_. C_A,X_ and C_B,X_ represent the concentrations of drug A and drug B, when used in combination to achieve x% drug effect. IC_X,A_ and IC_X,B_ represent the concentrations required for individual monotherapy to achieve the same x% effect. CI < 1 indicates synergy, CI = 1 demonstrates an additive effect, and CI > 1 represents antagonism^49^.

### Annexin V-binding assay for apoptosis

After cells were exposed to cisplatin and paclitaxel, each separately or in combination with SC66, apoptosis was measured using a FITC Annexin V Apoptosis Detection Kit (BD Pharmingen, Bedford, MA, USA) according to the manufacturer’s protocol. Collected cell suspensions were incubated with Annexin V for 15 min at room temperature in the dark and then analyzed by flow cytometry.

### Colony formation assay

Cells (300 per well) were cultured in 6-well plates in complete media overnight. After incubation, the culture media were replaced with fresh media containing SC66, cisplatin, or paclitaxel for 48 h. Treated cells were cultured in fresh media supplemented with 10% FBS for another 14 d for cell lines A2780, A2780CP70, A2780/V, and A2780/COL11A1, and 21 d for cell lines ES-2 and ES-2/CP. At the end of culturing, cells were stained with 0.01% crystal violet for 1 h at room temperature. The figures of colony formation studies have been shown in Supplementary Fig. 7.

#### Quantitative reverse transcriptase PCR

Total RNA (5 μg) was used as the template in cDNA synthesis reactions with random primers using Superscript III reverse transcriptase (Applied Biosystems). The resultant cDNAs were used (at a 1: 20 dilution) to detect the level of endogenous *COL11A1* mRNA expression by quantitative PCR (qPCR). Accurate quantification was achieved using standard curves generated by serially diluting a known quantity of RNA from an in vitro transcription reaction and performing TaqMan qPCR with the dilution along with the cell samples. Quantitative analysis of mRNA expression was performed the StepOne^TM^ Real-Time PCR System (ABI). The primers and TaqMan probes used for the analyses were designed using the manufacturer’s software Primer Express. The following primers were used: COL11A1 (HS01097664) and GAPDH (HS99999905). No-reverse-transcription (no-RT) control reactions were performed using 100 ng of total RNA from each individual sample as a template, to ensure that the amplification was not due to DNA contamination. No signal was detected in the no-RT controls. Target gene mRNA expression was assessed by real-time reverse transcriptase PCR. The reference gene *GAPDH* was used as the internal control for RNA quality. All of the quantitative analyses were performed in duplicate to assess the consistency of the results. The relative expression levels of the target gene, normalized to *GAPDH* expression, were calculated as Δ*C*_t_ = *C*_t_ (target) − *C*_t_ (GAPDH). The ratio of the number of copies of the target gene mRNA to the number of copies of *GAPDH* was then calculated as 2^−Ct^ × K (K = 10^6^, a constant).

### Plasmid construction and site-directed mutagenesis

*COL11A1* PCR product was cloned into the KpnI and XhoI sites of a pGL4 vector. The resultant construct was confirmed by DNA sequencing. *COL11A1* promoter deletion constructs COL11A1−541/+1, COL11A1−541/−203, and COL11A1−202/+1 were similarly generated using a COL11A1−541/+1 construct as a template, as previously described^22^.

### Luciferase reporter assays

Luciferase assays were performed 48 h after transfection, using a Dual-Luciferase Reporter Assay System (Promega). Normalized luciferase activity is reported as the ratio of luciferase activity to β-galactosidase activity, as previously described^22^.

### ChIP assays

Native protein–DNA complexes were crosslinked by treatment with 1% formaldehyde for 15 min and ChIP assays were performed as previously described^22^. Briefly, equal amounts of isolated chromatin were subjected to immunoprecipitation with anti-NF-YA, anti-c/EBPβ, and IgG monoclonal antibodies.

### Transwell® invasion assay

Invasion was examined in Transwell cell culture chambers using polycarbonate membranes with 8 μm pores (Costar, Cambridge, MA). The Transwell membranes coated with rat collagen I were placed as a barrier (60 µg/Transwell) on the upper side. Cells (5 × 10^4^) were placed in the upper chamber. The lower chamber contained 0.6 mL of medium containing fibronectin as a chemoattractant. Cells were allowed to invade for 24 h at 37 °C and 5% CO_2_. Non-migrated cells in the upper chamber were removed with a cotton swab and the filters were fixed in 95% ethanol and stained with 0.005% crystal violet for 1 h. The total number of cells that had migrated to the lower surface was counted using a fluorescence microscope (Olympus, Lake Success, NY). Ten contiguous fields were examined for each sample to obtain a representative number of cells that had invaded across the membrane. Each condition was assayed in triplicate. Data were shown as fold change in cell number of each group in comparison with A2780/V control group. *P*-value was compared between SC66 and control in different cell.

### Casein zymography analysis

To measure MMP3 activity, conditioned medium from treated cells was concentrated ~20-fold using Centricon-10 spin concentrators (Millipore, Billerica, MA, USA). Samples were quantified by Bradford analysis and equal amounts of protein were mixed with Laemmli sample buffer without reducing agents, incubated for 15 min at 37 °C, and separated on precast gradient SDS-polyacrylamide slab gels containing 1 mg/mL casein (Sigma). Following electrophoresis, the gels were placed in 2.5% Triton X-100 for 30 min, then incubated at 37 °C in 50 mM Tris–HCl, pH 7.4, containing 5 mM CaCl_2_ for 18 h. MMP3 bands were visualized by Coomassie blue staining and the level of MMP3 activity was quantified, as previously described^21^.

### Xenograft animal model

All animal procedures were reviewed and approved by the Institutional Animal Care and Use Committee at National Cheng Kung University. Female, 6-week-old NOD-SCID mice (National Cheng University Animal Center) were subcutaneously implanted in the rear flank with 1 × 10^6^ A2780/COL11A1 cells (100 μL). Tumor dimensions were measured two to three times per week and the volumes calculated as length (mm) × width (mm) × height (mm) × 0.52. Animal studies involving the measurement of tumor volume were usually performed with one control per experimental subject. The sample size for the experimental and control groups required to be sufficient to reject the null hypothesis that the population means of the experimental and control groups were equal with a power of 0.8 and type I error of 0.01. We estimated animal numbers using PS: Power and Sample Size Calculation, version 3.1.2 by William D. Dupont and Walton D. Plummer, Jr. (Vanderbilt University, Nashville, TN, USA). We estimated that the number of mice needed to assess tumor volume to be at least three for each of the control and experimental groups. Once tumors reached 20 mm^3^, mice were randomly assigned to one of nine groups (*n* = 4/group). Animals in each group received 200 μL of saline, cisplatin, paclitaxel, or SC66 by intraperitoneal injection. Treatment frequency was once per day for SC66 and once per 3 days for cisplatin and paclitaxel. Tumor growth, tumor imaging, and body weights were determined as previously described^50^. After 30 d, mice were killed using CO_2_ inhalation and the xenograft tumor tissues excised. Tumors were removed, weighed, fixed in 10% formalin, embedded in paraffin, and sectioned (4 μm) for histopathology and IHC. Paraffin sections of the tumors were stained with hematoxylin and eosin. An investigator (C.-C.C., a gynecologic pathologist) was responsible for interpreting the extent of cancer involvement in each organ. The anti-p-Akt, anti-Akt, anti-COL11A1, anti-Ki-67, and anti-caspase 3 primary antibodies were applied. Negative controls were treated with PBS. Intensely expressing p-Akt-positive human lung carcinoma, Akt-positive normal kidney tissues, COL11A1-positive normal hepatocytes, Ki-67-positive normal tonsil, and caspase 3-positive normal tonsil were used as positive controls. Staining intensity was scored as negative, weak, moderate, and strong staining (grade 0, 1, 2, and 3, respectively).

### Statistics

Data were analyzed using the Statistical Package for the Social Sciences software program, version 17.0 (SPSS, Inc., Chicago, IL, USA). Interval variables are shown as mean ± SEM; differences between groups were analyzed using the Mann–Whitney *U*-test. Frequency distributions between categorical variables were compared using Pearson’s *χ*^2^-test and Fisher’s exact method. Survival was estimated using the Kaplan–Meier method and results were compared by performing log-rank tests. *P*-values < 0.05 (two-sided) were considered to indicate statistical significance.

## Supplementary information

SC66 supplementary information

SC66 revised supplementary figure 1

SC66 revised supplementary figure 2

SC66 revised supplementary figure 3

SC66 revised supplementary figure 4

SC66 revised supplementary figure 5

SC66 revised supplementary figure 6

SC66 revised supplementary figure 7
