# Supplementary material for: Akt inhibitor SC66 promotes cell sensitivity to cisplatin in chemoresistant ovarian cancer cells through inhibition of COL11A1 expression
Source: Cell Death Dis. 2019 Apr 11;10(4):322. doi: 10.1038/s41419-019-1555-8 (PMC6459878; doi:10.1038/s41419-019-1555-8)
Supplement: Supplementary file 1 — SC66 supplementary information [file 41419_2019_1555_MOESM1_ESM.doc]

**Akt inhibitor SC66 promotes cell sensitivity to cisplatin in chemoresistant ovarian cancer cells through inhibition of COL11A1 expression**

Yi-Hui Wu^1^, Yu-Fang Huang^1^, Chien-Chin Chen^2, 3^ and Cheng-Yang Chou^1^

**Supplementary information**

**Supplementary Figure 1. Synergistic effect of SC66 combined with CDDP or PAC in A2780/V and A2780/COL11A1 cells.**

**Supplementary Figure 2. Tumor formation was not observed on 17 days after mice was injected with 1 × 10^6^ A2780/V cells.**

**Supplementary Figure 3. Representative IHC photos of Ki-67 and caspase 3 in ovarian tumor samples from mice treated with SC66 or vehicle controls.**

**Supplementary Figure 4.** **Cell viability of immortalized ovarian surface epithelial cells was measured by MTT assays. All experiments were performed in triplicate.**

**Supplementary Figure 5. MK-2206 did not regulate COL11A1 and PDK1 expression.**

**Supplementary Figure 6. Expression of p-Akt protein in tumors of ovarian cancer patients.**

**Supplementary Figure 7. Whole plates of colonies formation.**

**Supplementary Table 1. Immunohistochemistrical phosphor-Akt** (p-Akt) expression and patient demographics (n=230).

|  |  | |  | p-Akt staining | |  |
| --- | --- | --- | --- | --- | --- | --- |
| Variable | | | N | Low | High | *p* |
| **All patients** | | |  | N=187 | N=43 |  |
| Age (year) | | ≤ 52 | 115 | 91 (79.1) | 24 (20.9) | 0.398 |
|  |  | > 52 | 115 | 96 (83.5) | 19 (16.5) |  |
| FIGO Stage | | Early | 89 | 76 (85.4) | 13 (14.6) | 0.206 |
|  |  | Advanced | 141 | 111 (78.7) | 30 (21.3) |  |
| Histology | | Serous | 129 | 107 (82.9) | 22 (17.1) | 0.471 |
|  |  | Non-serous | 101 | 80 (79.2) | 21 (20.8) |  |
| Grade | | 1 & 2 | 92 | 82 (89.1) | 10 (10.9) | 0.013 |
|  |  | 3 | 138 | 105 (76.1) | 33 (23.9) |  |
| Residual nodules | | < 1 cm | 175 | 146 (83.4) | 29 (16.6) | 0.141 |
|  |  | ≥ 1 cm | 55 | 41 (74.5) | 14 (25.5) |  |
| First-line chemotherapy | | None | 24 | 22 (91.7) | 2 (8.3) | 0.101 |
|  |  | Paclitaxel/platinum | 168 | 131 (78.0) | 37 (22.0) |  |
|  |  | Other/platinum | 38 | 34 (89.5) | 4 (10.5) |  |
| Response to chemotherapy (n=206) | | CR & PR | 167 | 136 (81.4) | 31 (18.6) | 0.521 |
|  |  | SD & PD | 39 | 30 (76.9) | 9 (23.1) |  |
| PFI | | < 6 months | 60 | 44 (73.3) | 16 (26.7) | 0.065 |
|  |  | ≥ 6 months | 170 | 143 (84.1) | 27 (15.9) |  |

Data was analyzed by X^2^ test or Fisher’s exact test.

Non-serous type included clear cell (n=39), endometrioid (n=33), mucinous (n=25) and others (n=4).

Early stage included stage I (n=72) and stage II (n=17); advanced stage included stage III (n=121) and stage IV (n=20).

Abbreviation: FIGO, International Federation of Gynecology and Obstetrics; CR, complete response; PR, partial response; SD, stable disease; PD, progressive disease; PFI, progression-free interval.
