# Supplementary material for: Akt inhibitor SC66 promotes cell sensitivity to cisplatin in chemoresistant ovarian cancer cells through inhibition of COL11A1 expression
Source: Cell Death Dis. 2019 Apr 11;10(4):322. doi: 10.1038/s41419-019-1555-8 (PMC6459878; doi:10.1038/s41419-019-1555-8)
Supplement: Supplementary file 2 — SC66 revised supplementary figure 1 [file 41419_2019_1555_MOESM2_ESM.ppt]

## Slide 1
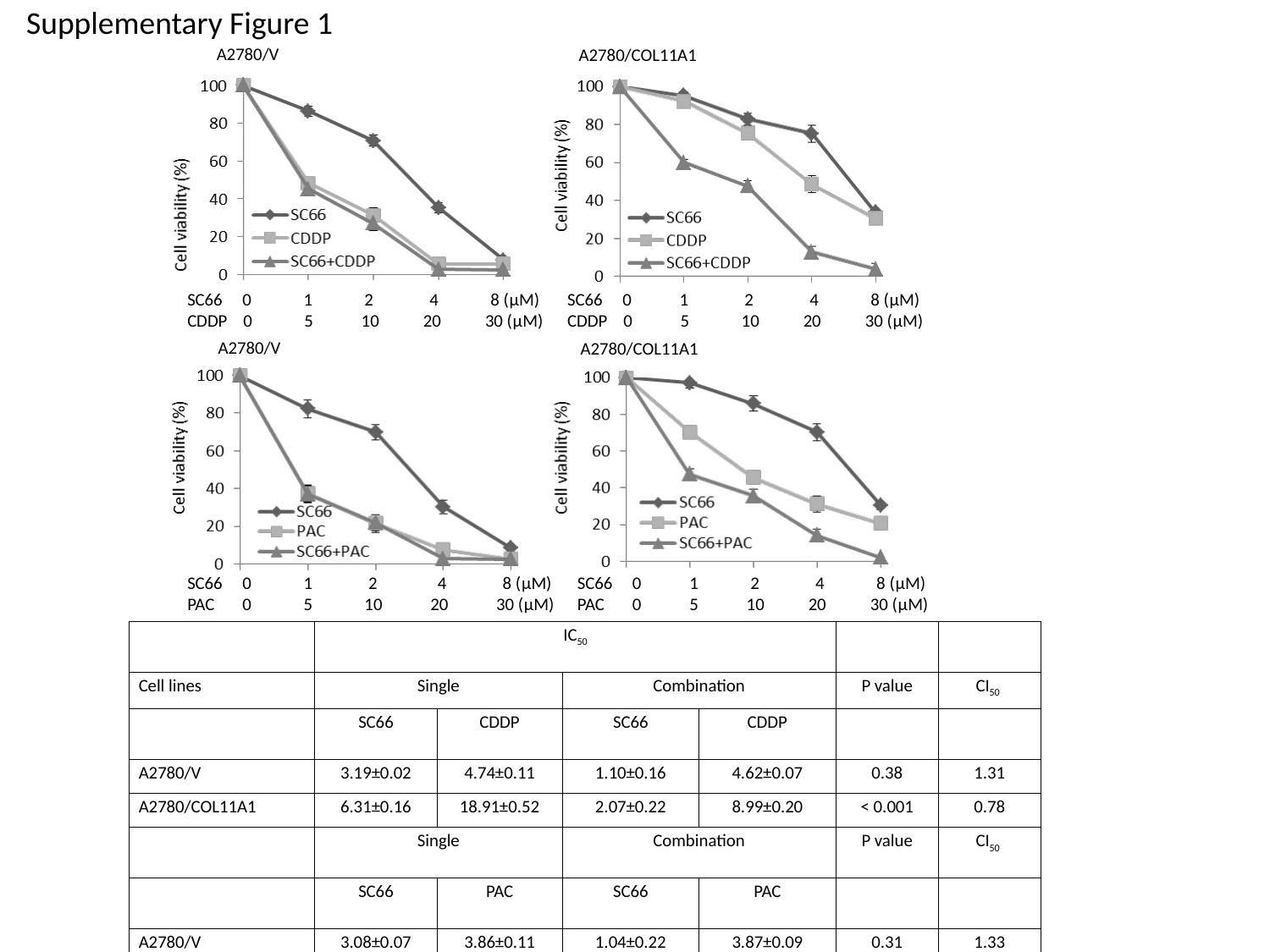

Supplementary Figure 1
A2780/V
A2780/COL11A1
SC66 0 1 2 4 8 (μM)
CDDP 0 5 10 20 30 (μM)
SC66 0 1 2 4 8 (μM)
CDDP 0 5 10 20 30 (μM)
A2780/V
A2780/COL11A1
SC66 0 1 2 4 8 (μM)
PAC 0 5 10 20 30 (μM)
SC66 0 1 2 4 8 (μM)
PAC 0 5 10 20 30 (μM)
| | IC50 | | | | | |
| --- | --- | --- | --- | --- | --- | --- |
| Cell lines | Single | | Combination | | P value | CI50 |
| | SC66 | CDDP | SC66 | CDDP | | |
| A2780/V | 3.19±0.02 | 4.74±0.11 | 1.10±0.16 | 4.62±0.07 | 0.38 | 1.31 |
| A2780/COL11A1 | 6.31±0.16 | 18.91±0.52 | 2.07±0.22 | 8.99±0.20 | < 0.001 | 0.78 |
| | Single | | Combination | | P value | CI50 |
| | SC66 | PAC | SC66 | PAC | | |
| A2780/V | 3.08±0.07 | 3.86±0.11 | 1.04±0.22 | 3.87±0.09 | 0.31 | 1.33 |
| A2780/COL11A1 | 6.04±0.08 | 7.70±0.79 | 1.02±0.08 | 4.70±0.05 | 0.02 | 0.77 |
