# Supplementary figures and images for: Akt inhibitor SC66 promotes cell sensitivity to cisplatin in chemoresistant ovarian cancer cells through inhibition of COL11A1 expression
Source: Cell Death Dis. 2019 Apr 11;10(4):322. doi: 10.1038/s41419-019-1555-8 (PMC6459878; doi:10.1038/s41419-019-1555-8)

## Slide 1
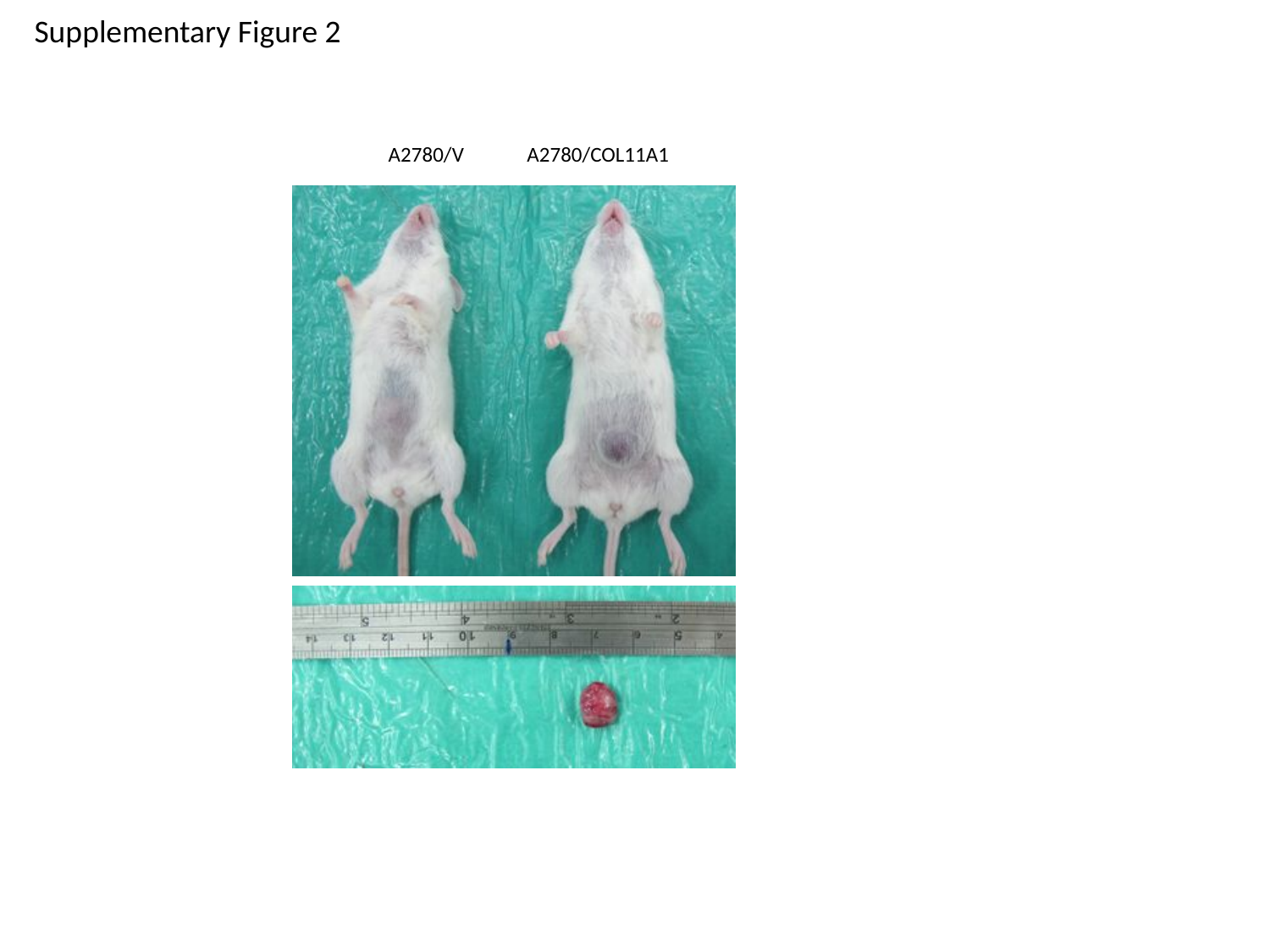

Supplementary Figure 2
A2780/V A2780/COL11A1

Supplement: Supplementary file 3 — SC66 revised supplementary figure 2 [file 41419_2019_1555_MOESM3_ESM.ppt]

## Slide 1
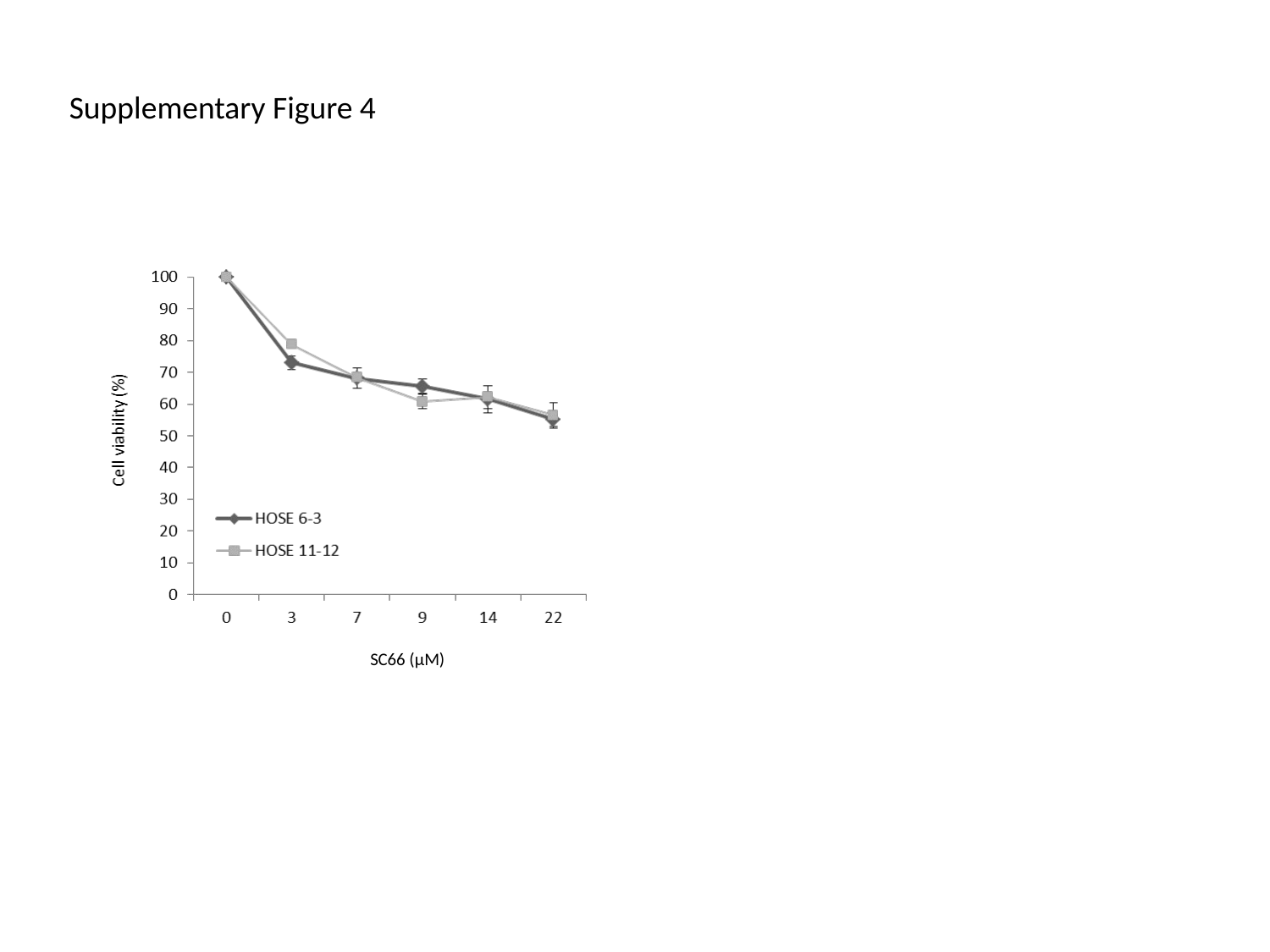

Supplementary Figure 4
SC66 (μM)

Supplement: Supplementary file 5 — SC66 revised supplementary figure 4 [file 41419_2019_1555_MOESM5_ESM.ppt]

## Slide 1
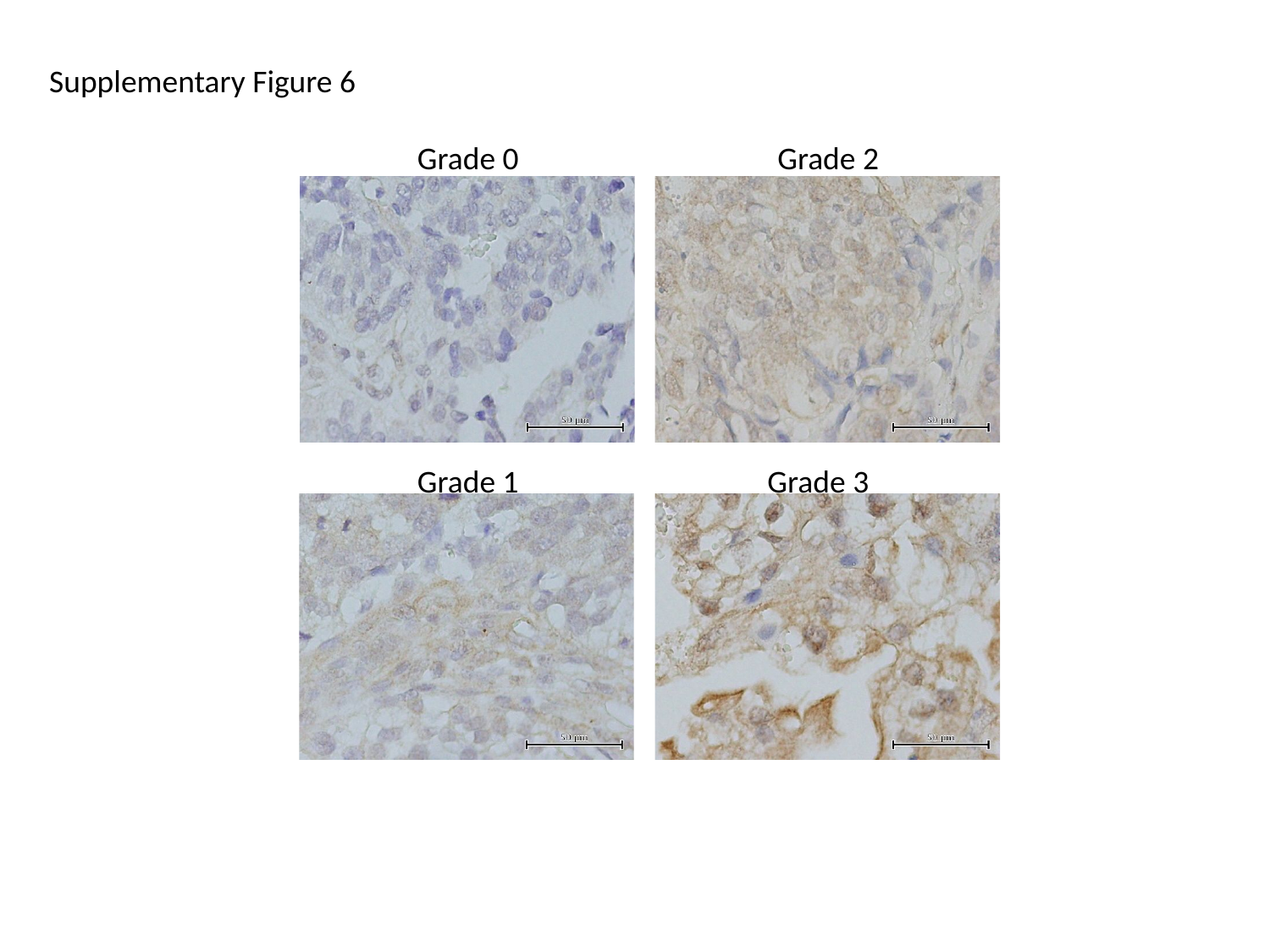

Supplementary Figure 6
Grade 0
Grade 2
Grade 1
Grade 3

Supplement: Supplementary file 7 — SC66 revised supplementary figure 6 [file 41419_2019_1555_MOESM7_ESM.ppt]

## Slide 1
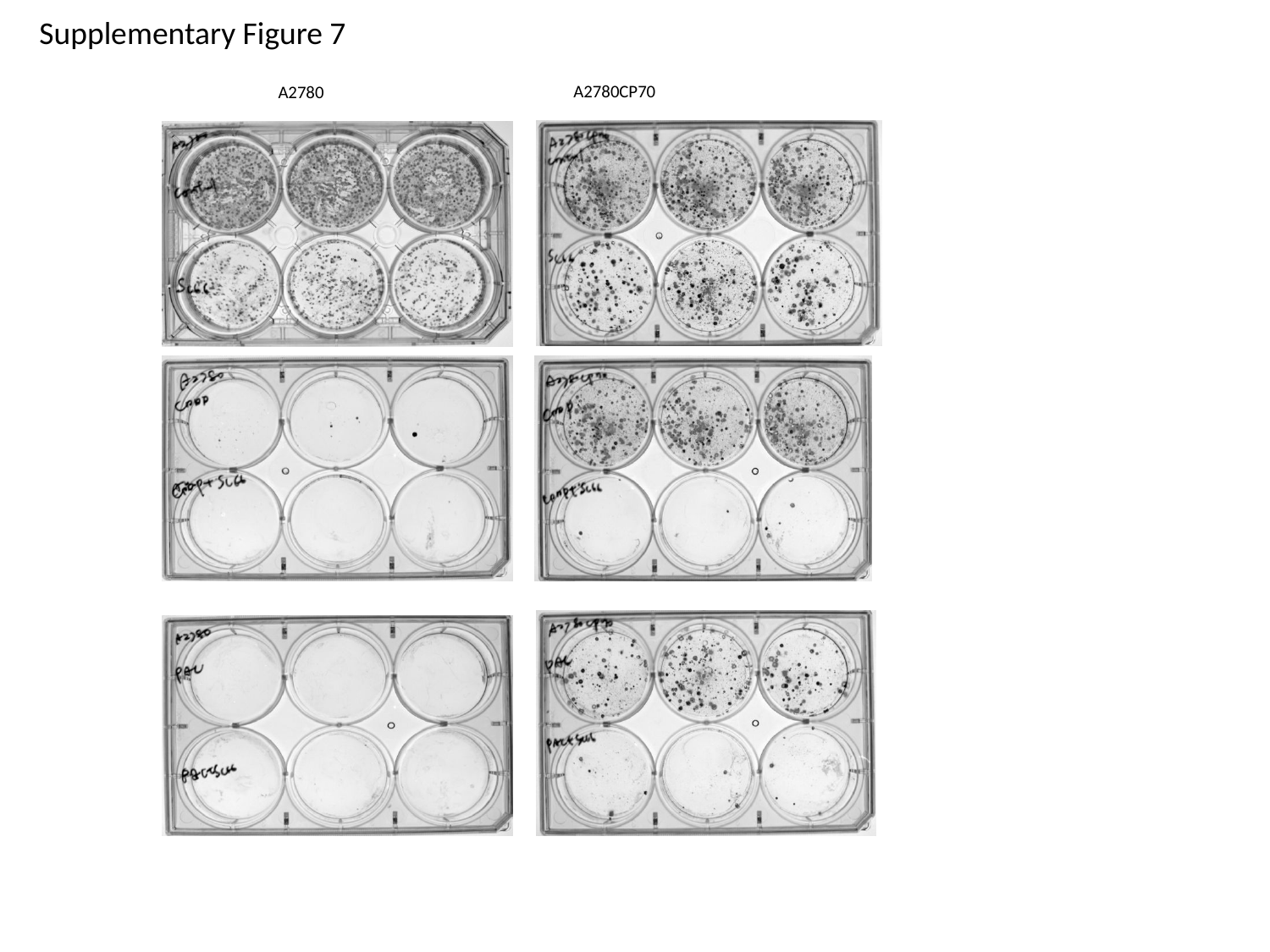

Supplementary Figure 7
A2780CP70
A2780

## Slide 2
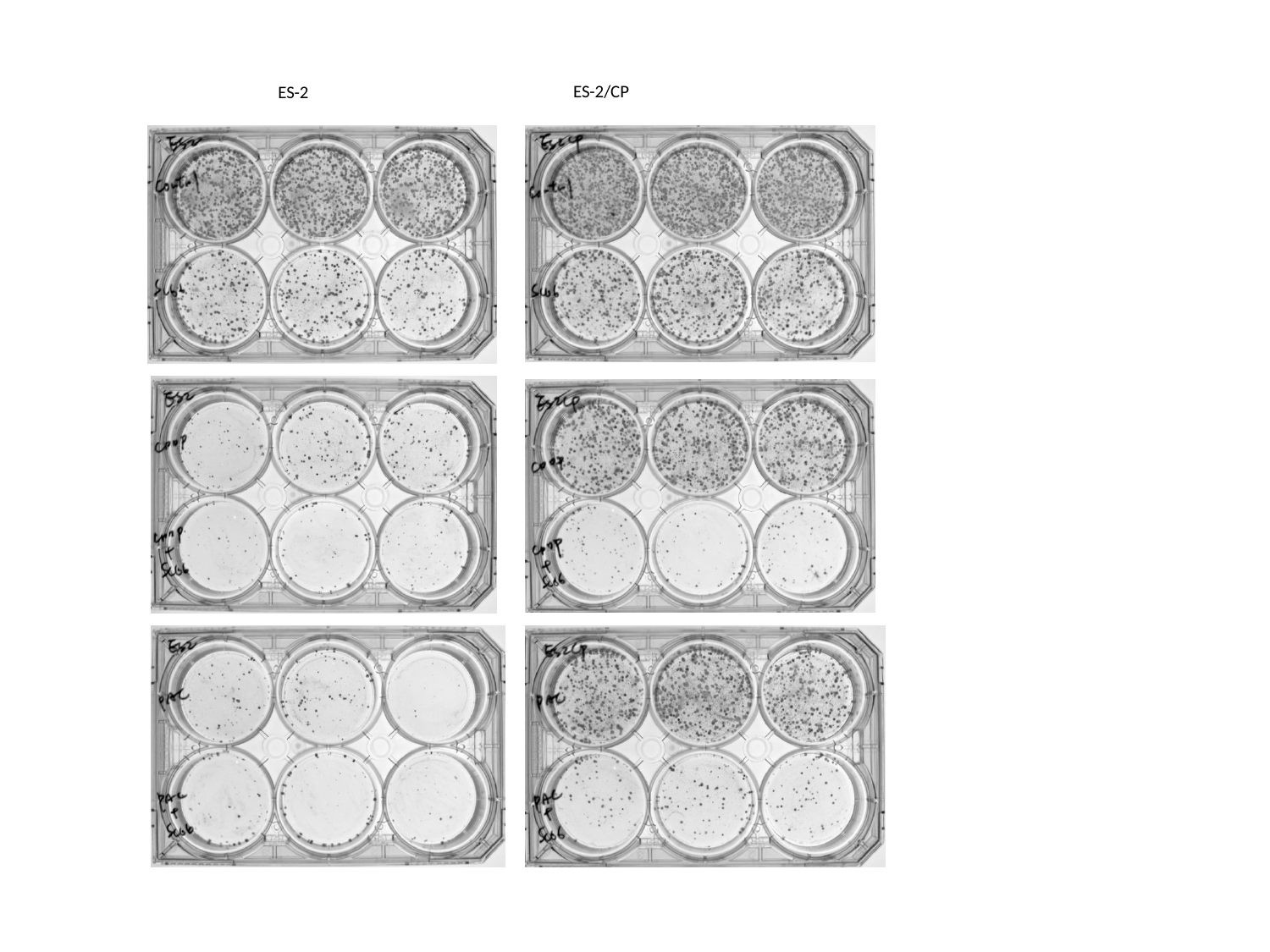

ES-2/CP
ES-2

## Slide 3
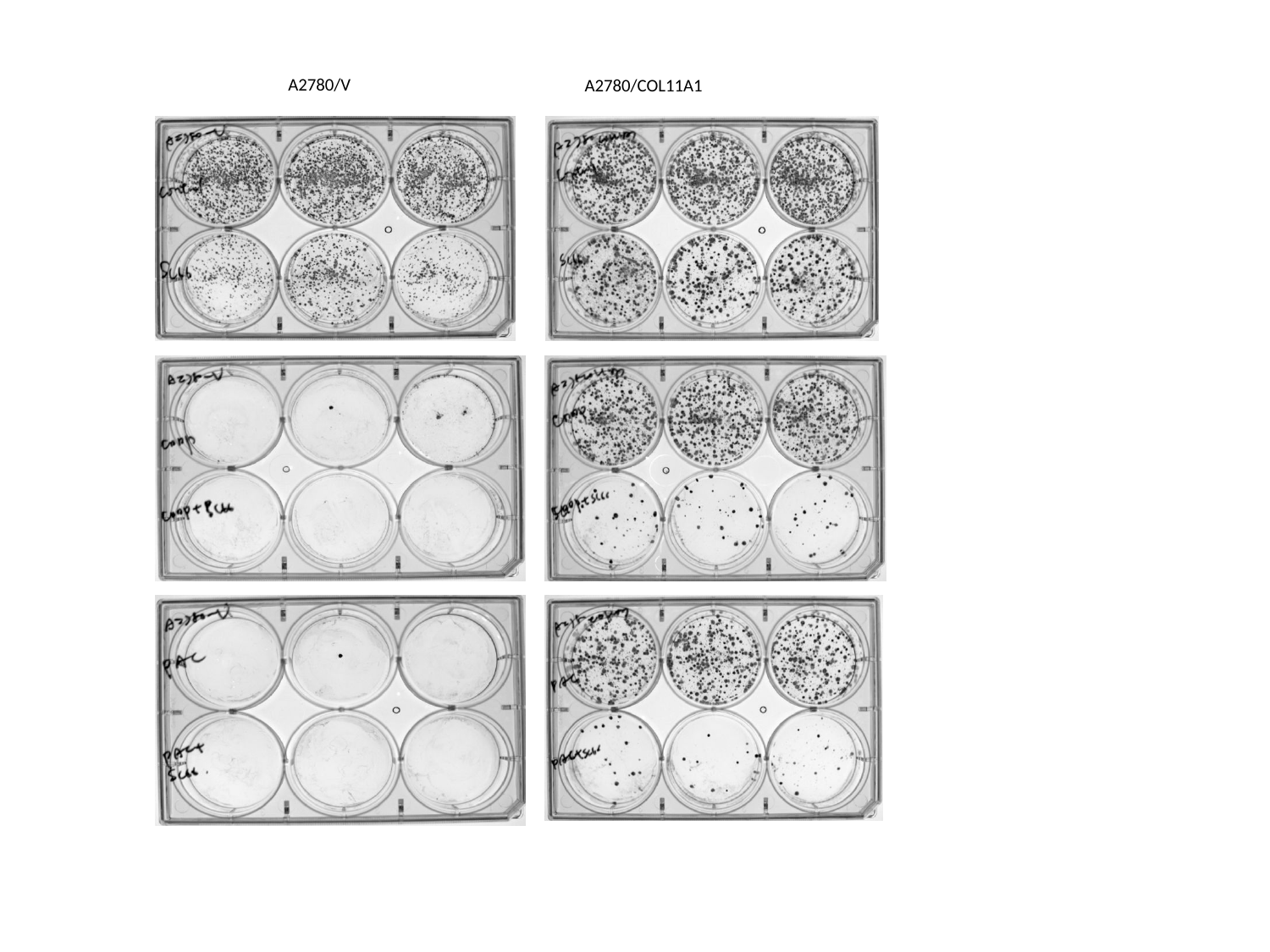

A2780/V
A2780/COL11A1

Supplement: Supplementary file 8 — SC66 revised supplementary figure 7 [file 41419_2019_1555_MOESM8_ESM.ppt]
