# Supplementary material for: Akt inhibitor SC66 promotes cell sensitivity to cisplatin in chemoresistant ovarian cancer cells through inhibition of COL11A1 expression
Source: Cell Death Dis. 2019 Apr 11;10(4):322. doi: 10.1038/s41419-019-1555-8 (PMC6459878; doi:10.1038/s41419-019-1555-8)
Supplement: Supplementary file 4 — SC66 revised supplementary figure 3 [file 41419_2019_1555_MOESM4_ESM.ppt]

## Slide 1
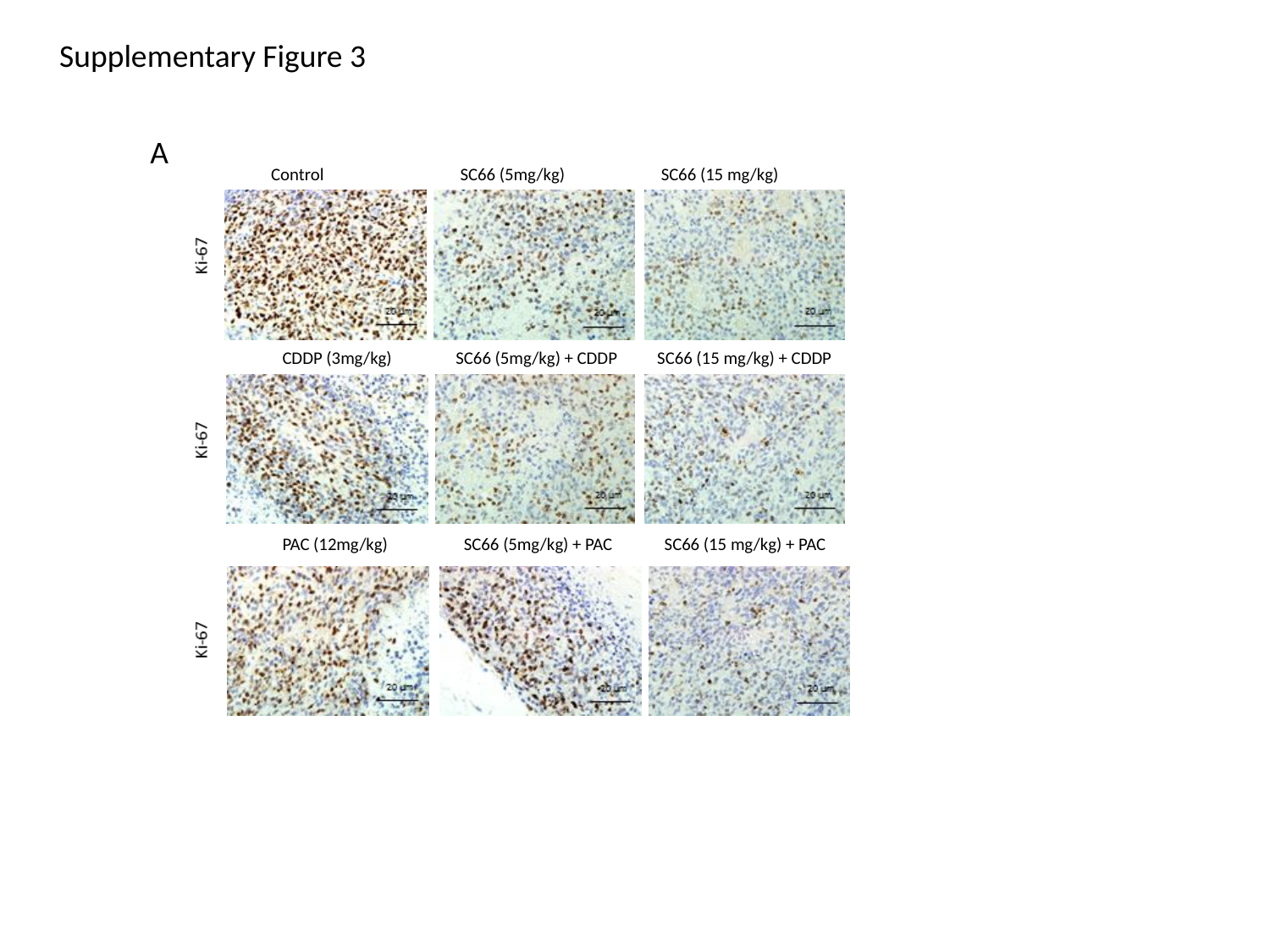

Supplementary Figure 3
A
Control SC66 (5mg/kg) SC66 (15 mg/kg)
CDDP (3mg/kg) SC66 (5mg/kg) + CDDP SC66 (15 mg/kg) + CDDP
PAC (12mg/kg) SC66 (5mg/kg) + PAC SC66 (15 mg/kg) + PAC

## Slide 2
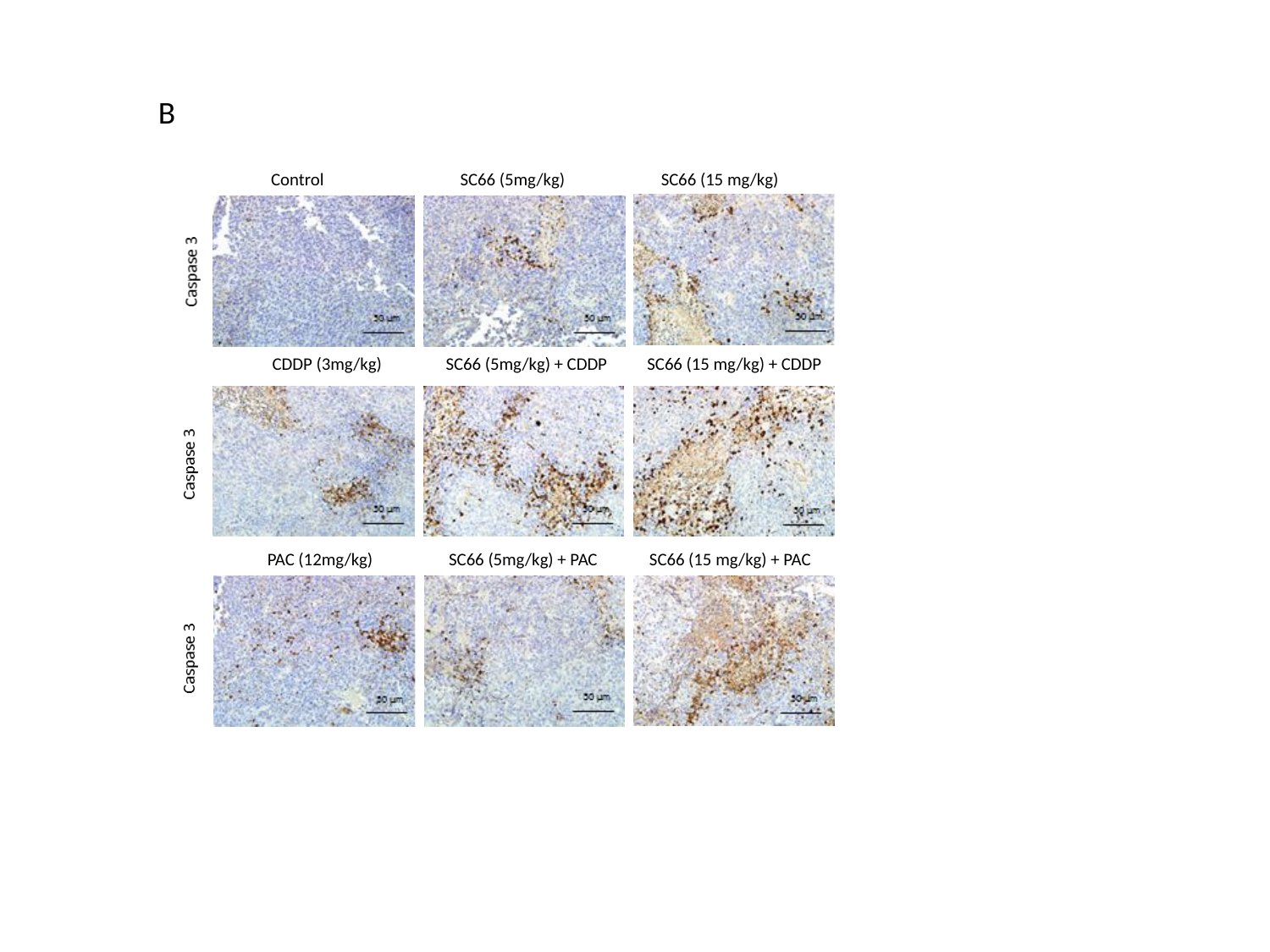

B
Control SC66 (5mg/kg) SC66 (15 mg/kg)
CDDP (3mg/kg) SC66 (5mg/kg) + CDDP SC66 (15 mg/kg) + CDDP
PAC (12mg/kg) SC66 (5mg/kg) + PAC SC66 (15 mg/kg) + PAC
