# Supplementary material for: Akt inhibitor SC66 promotes cell sensitivity to cisplatin in chemoresistant ovarian cancer cells through inhibition of COL11A1 expression
Source: Cell Death Dis. 2019 Apr 11;10(4):322. doi: 10.1038/s41419-019-1555-8 (PMC6459878; doi:10.1038/s41419-019-1555-8)
Supplement: Supplementary file 6 — SC66 revised supplementary figure 5 [file 41419_2019_1555_MOESM6_ESM.ppt]

## Slide 1
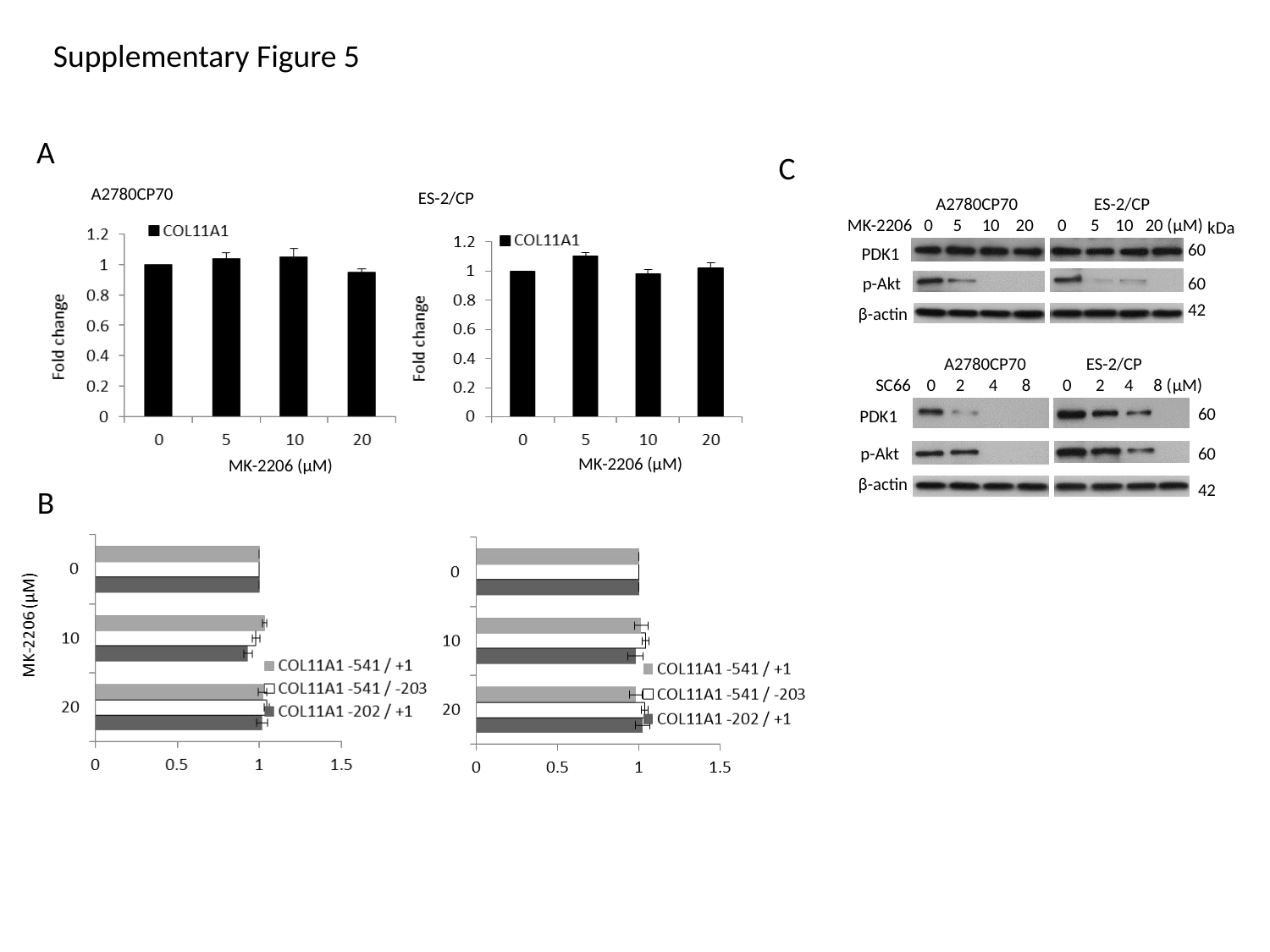

Supplementary Figure 5
A
C
A2780CP70
ES-2/CP
 A2780CP70 ES-2/CP
MK-2206 0 5 10 20 0 5 10 20 (μM)
kDa
60
PDK1
p-Akt
60
42
β-actin
 A2780CP70 ES-2/CP
SC66 0 2 4 8 0 2 4 8 (μM)
60
PDK1
60
p-Akt
MK-2206 (μM)
MK-2206 (μM)
β-actin
42
B
